# Auxin-Responsive R2R3-MYB Transcription Factors HcMYB1 and HcMYB2 Activate Volatile Biosynthesis in *Hedychium coronarium* Flowers

**DOI:** 10.3389/fpls.2021.710826

**Published:** 2021-08-03

**Authors:** Yanguo Ke, Farhat Abbas, Yiwei Zhou, Rangcai Yu, Yanping Fan

**Affiliations:** ^1^The Research Center for Ornamental Plants, College of Forestry and Landscape Architecture, South China Agricultural University, Guangzhou, China; ^2^College of Economics and Management, Kunming University, Kunming, China; ^3^College of Life Sciences, South China Agricultural University, Guangzhou, China; ^4^Guangdong Key Laboratory for Innovative Development and Utilization of Forest Plant Germplasm, South China Agricultural University, Guangzhou, China

**Keywords:** *Hedychium coronarium*, floral scent, auxin, MYB transcription factors, biosynthesis

## Abstract

Auxin, an important plant hormone, induces the biosynthesis of various secondary metabolites by modulating the expression of auxin-responsive genes. In the ornamental plant *Hedychium coronarium*, linalool and methyl benzoate are biosynthesized by the terpene synthase (TPS) HcTPS5 and the benzoic/salicylic acid methyltransferase (BSMT) HcBSMT2, respectively. However, the transcriptional regulation of this process remains unclear. Here, we identified and functionally characterized the R2R3-MYB transcription factors HcMYB1 and HcMYB2 in regulating the biosynthesis of these floral aroma compounds. *HcMYB1* and *HcMYB2* are specifically expressed in flowers, their expression is correlated with the emission of volatile compounds in flowers, and is induced by auxin. Moreover, HcMYB1 and HcMYB2 interact with the *HcBSMT2* promoter region. HcMYB2 activates the expression of the linalool synthase gene *HcTPS5*. In flowers with *HcMYB1* or *HcMYB2* silenced, the levels of floral scent compounds were significantly reduced, and *HcBSMT2* and *HcTPS5* were downregulated compared with the wild type. Moreover, HcMYB1 form protein-protein interaction with key scent-related HcIAA4 protein to regulate floral aroma production. Taken together, these results indicate that HcMYB1 and HcMYB2 play crucial roles in regulating the formation of scent compounds in *Hedychium coronarium (H. coronarium)* flowers in response to auxin signaling.

## Introduction

Floral scent compounds are among the most important secondary metabolites in plants and comprise three major groups based on their origins: terpenoids, phenylpropanoids/benzenoids, and fatty acid derivatives (Dudareva et al., 2013; Muhlemann et al., 2014; Abbas et al., 2017). These compounds are attractive not only to humans but also to pollinators that facilitate fertilization, and thus they play key roles in plant evolution and the plant lifecycle (Raguso, 2009). The anti-herbivore or antimicrobial activity of volatiles released from flowers protects the vulnerable reproductive parts of the plant against pathogen attack (Dudareva et al., 2006). Floral scent also is an important trait that increases the aesthetic values of ornamental plants to humans (Dudareva et al., 2006; Pichersky and Dudareva, 2007), and scented compounds derived from flowers are widely used as flavorings, in cosmetics and perfumes, and as medicinal substances (Gershenzon and Dudareva, 2007; Muhlemann et al., 2014). However, notwithstanding the importance of floral scents to both plant biology and industry, little is known about the transcriptional regulation of this process.

MYB transcription factors are important regulators of the biosynthesis of plant secondary metabolites, such as phenylpropanoids (Liu L. et al., 2015; Zhou and Memelink, 2016; Ramya et al., 2017). MYB proteins possess two regions: a conserved MYB DNA-binding domain at the N-terminus and a diverse modulator region at the C-terminus that is responsible for their regulatory activity. MYB transcription factors (TFs) are classified into four subunits/groups based on the number of adjacent repeats in the DNA-binding domains: the R2R3-, 1R-, 3R-, and 4R-MYBs (Dubos et al., 2008). Most MYB TFs involved in regulating secondary metabolite biosynthesis in flowers belong to the R2R3-MYB family (Ramya et al., 2017). To date, however, only a few MYB TFs identified from floral scent model species, i.e., snapdragon (*Antirrhinum majus*) and petunia (*Petunia* spp.) have been shown to regulate the expression of structural genes related to volatile biosynthetic pathways. The R2R3-MYB TFs ODORANT1 (ODO1) and EMISSION OF BENZENOID II (EOBII) regulate volatile biosynthesis genes in the benzenoid/phenylpropanoid pathway in petunia (Verdonk et al., 2005; Spitzer-Rimon et al., 2012). In snapdragon, the MYBs AmMYB305 and AmMYB340 are involved in regulating the volatile phenylpropanoid/benzenoid metabolic pathway (Uimari and Strommer, 1997; Shin et al., 2002). In addition, the R2R3-MYB TF FaEOBII regulates the production of the volatile eugenol in ripe strawberry (*Fragaria* × *ananassa*) receptacles by activating the expression of *CINNAMYL ALCOHOL DEHYDROGENASE* (*FaCAD1*) and *EUGENOL SYNTHASE* (*FaEGS2*) (Medina-Puche et al., 2015). In the *Cymbidium* orchid cultivar “Sael Bit,” *CsMYB1* is highly expressed in floral organs and is involved in regulating the biosynthesis of floral volatiles such as polyacrylate and 2-methyl butyraldehyde in petals (Ramya et al., 2019).

Floral and fruit volatiles are also regulated by the essential plant hormone auxin, which induces the biosynthesis of numerous secondary metabolites by regulating the expression of auxin-responsive genes (Zhou and Memelink, 2016). Treatment with exogenous auxin increases the emission of the volatile compound linalool in apple (*Malus domestica*) scions (Kim et al., 2011) and modifies the quantity of fruit flavor compounds. In strawberry, auxin treatment enhances the aggregation of phenolic volatiles such as 2-phenylethanol, phenylacetaldehyde, and methyl benzoate, and inhibits the production of benzyl cyanide, 2-isobutylthiazole, 1-hexanol, and 1-nitro-2-phenylethane (Wu et al., 2018). Exogenous auxin treatment also modifies the expression of several key genes associated with the biosynthetic pathways of scent volatiles, including *PHENYL ALDEHYDE REDUCTASE 1* (*SlPAR1*), *SlPAR2*, and *SlSAMT1*, in tomato (*Solanum lycopersicum*) (Wu et al., 2018). In grapefruit (*Citrus* × *paradisi*), auxin treatment influences sugar accumulation in various ways, as well as the accumulation of volatile compounds and the expression of aroma-related genes (Jia et al., 2017). Many MYB TF genes respond to auxin signalings, such as the *Arabidopsis thaliana* genes *AtMYB44*, *AtMYB77*, and *AtMYB108* (Shin et al., 2007; Dubos et al., 2010) and ten R2R3-MYB genes in cassava (*Manihot esculenta*) (Liao et al., 2016). Nevertheless, how auxin is involved in regulating the phenylpropanoid and terpenoid biosynthetic pathway via MYB TFs was not known.

*Hedychium coronarium* is a perennial herb of the Zingiberaceae family that is cultivated as a cut flower, garden plant, and medicinal plant and for aromatic oil production. At blooming, *H. coronarium* flowers emit large amounts of volatile compounds, including the monoterpenes linalool and (*E*,Z)-β-ocimene, and benzenoids such as methyl benzoate (Fan et al., 2003, 2007; Li and Fan, 2011; Lan et al., 2013; Yue et al., 2015). We previously identified several structural genes in the *H. coronarium* volatile biosynthetic pathway, including genes encoding terpene synthases (TPSs) and benzoic/salicylic acid methyltransferase (BSMT). A total of 12 *HcBSMT* and 62 *HcTPS* genes were found in *H. coronarium*. *HcBSMT2* specifically expressed in flowers, its expression level was enormously high among all *HcBSMT* genes and correlated with flower development (Supplementary Figure 6). Likewise, the expression values of *HcTPS3* and *HcTPS5* were tremendously high among all the *HcTPSs* and specifically expressed in *H. coronarium* flowers (Supplementary Figure 7). Moreover, their expression pattern positively correlated with flower development as well as with the emission of monoterpenes, and their encoded enzymes localize to plastids (Yue et al., 2015). Our functional characterization indicated that HcTPS3 functions in (*E*)-β-ocimene production, HcTPS5 functions in linalool production, and HcBSMT2 functions in methyl benzoate production (Yue et al., 2015, 2021). Analysis of previously generated RNA-seq data showed that six *HcMYB* family members were clustered in a group involved in regulating secondary metabolism. The expression levels of these MYB family members were analyzed in different tissues (flowers, bracts, leaves, and rhizomes). The RNA-sequence data showed that among six *HcMYBs*, *HcMYB1*, and *HcMYB2* were highly flower-specific and the abundance of their transcripts correspond with the flower development as well as with the emission of floral volatile contents. The relative transcript abundance of six *HcMYB* family members has been provided in Supplementary Figure 3. However, the hormone-responsive transcriptional regulation of these genes has not been elucidated. Furthermore, we comprehensively analyzed *Aux/IAA* genes in *H. coronarium* genome. The genome−wide analysis and characterization of *Aux/IAA* genes reveal the potential role of *HcIAA2* and *HcIAA4* in floral aroma production in *H. coronarium* (Ke et al., 2019).

In the current study, we functionally characterized two R2R3-MYB TF genes (*HcMYB1* and *HcMYB2*) that are expressed in an auxin-responsive manner specifically in flowers. These MYB TFs regulate phenylpropanoid/benzenoid and terpenoid biosynthesis specifically in *H. coronarium* flowers by activating *HcBSMT2* and *HcTPS5* expression. Furthermore, the interaction of HcMYB1 with key scent-related auxin protein (HcIAA4) was revealed via yeast two-hybrid (Y2H) assay and bimolecular fluorescence complementation (BiFC) assays. These findings shed light on the mechanism underlying the emission of floral scent compounds in *H. coronarium*.

## Results

### Identification and Characterization of Scent-Related R2R3-MYB Family Members

**FIGURE 1:**
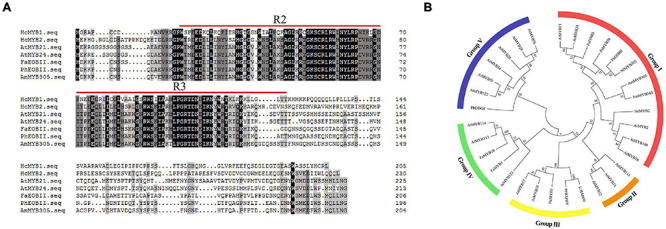
Multiple sequence alignment and phylogenetic analysis of R2R3-MYB proteins. **(A)** Multiple sequence alignment of HcMYB1 and HcMYB2 with other related MYBs from different plants. The conserved R2 and R3 motifs are underlined. Multiple sequence alignment was performed using Clustal Ω and edited with GeneDoc. **(B)** Phylogenetic analysis of HcMYB1 and HcMYB2 together with selected R2R3-MYB proteins from different plants. Amino acid sequence alignment was performed using Clustal Ω, and the tree was constructed using the NJ method in MEGA X. The bootstrap values were set to 1000. The accession numbers for the protein sequences are listed in Supplementary Table 3. The sequence info of HcMYB1 and HcMYB2 is given in Supplementary Material.

In a previous transcriptomic analysis, we identified a clade of genes whose expression rose throughout flower development and with increasing floral scent emissions (Yue et al., 2015). Among these genes, *HcMYB1* and *HcMYB2* are specifically expressed in flowers. The full-length complementary DNA (cDNA) sequences of *HcMYB1* and *HcMYB2* contain open reading frames (ORFs) of 618 and 693 bp, encoding polypeptides of 205 and 230 amino acid residues with molecular masses of 23.26 and 24.68 kDa, respectively. Analysis of the predicted protein sequences of *HcMYB1* and *HcMYB2* revealed the presence of 2R and 3R repeat signatures at the N-termini: these features of R2R3 DNA-binding MYB proteins (Figure 1A) are essential for their interactions with regulatory sequences in the promoters of their target genes (Kranz et al., 1998; Dubos et al., 2010; Medina-Puche et al., 2014).

We performed a phylogenetic analysis of HcMYB1 and HcMYB2 compared to R2R3-MYBs involved in secondary metabolism in other plant species. HcMYB1 and HcMYB2 clustered into different groups (Figure 1B). HcMYB1 belongs to Group III and shares high amino acid homology with AtMYB77 and AtMYB44 (Aharoni et al., 2001; Shin et al., 2007; Jaradat et al., 2013). In contrast, HcMYB2 belongs to Group I, whose members include AmMYB305, AmMYB340 (*A. majus*), AtMYB24 (*A. thaliana*), AtMYB21 (*A. thaliana*), PhEOBII (*Petunia* × *hybrida*), FaEOBII (*Fragaria* × *ananassa*) NlMYB305 (*Nicotiana langsdorffii*), and PsMYB26 (*Pisum sativum*) (Uimari and Strommer, 1997; Shin et al., 2002; Li et al., 2006b; Liu et al., 2009; Spitzer-Rimon et al., 2010; Medina-Puche et al., 2015). The two proteins clustered into different groups and may have different functions and/or operate through different pathways to take part in floral volatile production. Furthermore, to interrogate the evolutionary relationship of six HcMYB family members with Arabidopsis MYBs, a phylogenetic tree was built. The phylogenetic analysis revealed that all MYB proteins can be clustered into five different groups (G A–G F). HcMYB1 was clustered into group G B, HcMYB2/6/5 belongs to group G D, while HcMYB3/4 was grouped into G A (Supplementary Figure 1).

### *HcMYB1* and *HcMYB2* Are Expressed During Flower Development and in Response to Auxin

**FIGURE 2:**
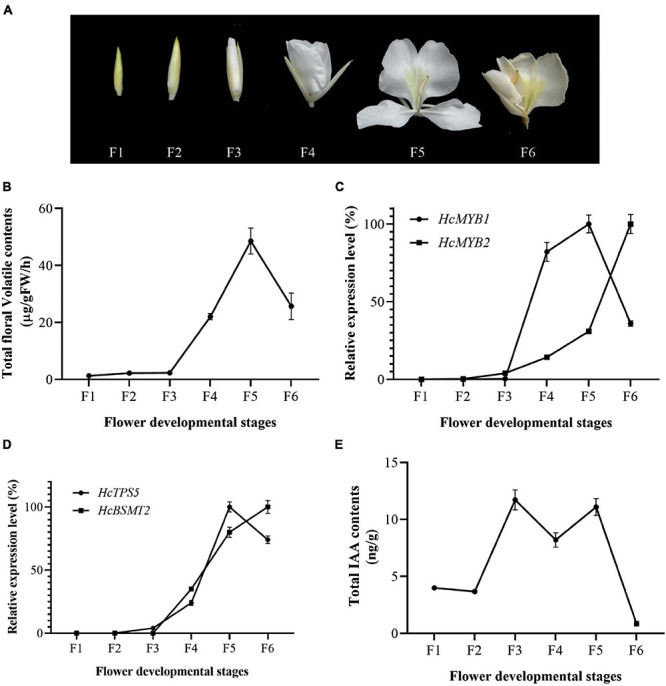
Changes in gene expression, IAA, and volatiles over the course of flower development **(A)** Pictorial view of different stages in floral development in *H. coronarium*. **(B)** Total floral volatile contents during flower development (F1–F6). **(C)** Expression analysis of *HcMYB1* and *HcMYB2* during different stages of flower development. **(D)** Relative expression levels of *HcTPS5* and *HcBSMT2*. **(E)** Endogenous auxin contents during flower development. Bars represent the means ± SD (*n* = 3–5).

The accumulation of floral volatiles increases as flower development proceeds (Yue et al., 2015; Abbas et al., 2019). To study this process, we divided the flower development process into six stages (Figure 2A). The emission of floral volatiles was low during the bud period (F1 and F2), substantially increased beginning at the initial flowering stage (F3), peaked during the full-bloom stage (F4 and F5), and declined at the senescence stage (F6) (Figure 2B).

To examine the relationship between *HcMYB1* and *HcMYB2* and key volatile biosynthesis genes (*HcTPS5* and *HcBSMT2*) involved in floral volatile contents, we measured the expression levels of these genes. *HcMYB1* and *HcMYB2* transcript levels were low during early flower development and substantially increased thereafter. *HcMYB1* expression peaked at the full-bloom stage (F4–F5) and decreased at the senescence stage (F6), whereas *HcMYB2* was most strongly expressed at F6 (Figure 2C). We detected similar expression patterns for *HcTPS5* and *HcBSMT2* during flower development (Figure 2D). Moreover, the expression levels of *HcMYB1* and *HcMYB2* were positively correlated with the emissions of floral volatiles; this correlation was highly significant for *HcMYB1* (Supplementary Figure 2). Moreover, HcMYB1 showed a highly significant correlation with the emission of linalool contents (Supplementary Figure 3). These results suggest that these genes play important roles in floral scent formation in *H. coronarium*.

**FIGURE 3:**
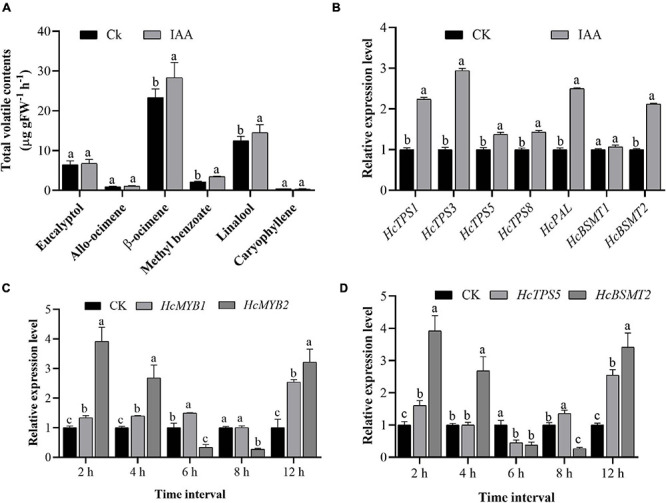
Exogenous auxin treatment influences the expression of *HcMYB1*, *HcMYB2*, and key volatile biosynthesis genes. **(A)** The emission of major floral volatile compounds in *H. coronarium* after auxin treatment. **(B)** Relative expression levels of key volatile biosynthesis genes after auxin treatment. **(C,D)** Expression patterns of *HcMYB1/2* and key structural genes (*HcTPS5* and *HcBSMT2*) in response to auxin treatment. Bars represent the means ± SD (*n* = 3–5). Different letters on bars indicate statistically significant differences (*P* < 0.05).

Auxin plays a crucial role throughout flower development (Krizek, 2011; Ke et al., 2018). We, therefore measured total auxin levels in *H. coronarium* during flower development (F1–F6). The total auxin contents were low during F1 and F2, peaked at F3–F5, dropped slightly at F4, and declined further at F6 (Figure 2E). The emission of total floral volatiles was correlated with indole-3-acetic acid (IAA) contents, suggesting that auxin might play a crucial role in the biosynthesis of these compounds. Under IAA treatment, the contents of the major floral volatiles ocimene, linalool, and methyl benzoate increased by 16, 17, and 20%, respectively, compared to those in control flowers not treated with IAA (CK) (Figure 3A). Moreover, the expression of key structural volatile biosynthesis genes (*HcTPS1*, *HcTPS3*, *HcTPS5*, *HcTPS8*, *HcPAL*, and *HcBSMT2*) was upregulated by this treatment (Figure 3B).

To characterize the expression levels of *HcMYB1* and *HcMYB2* in response to IAA treatment, we performed qRT-PCR analysis. *HcMYB1* and *HcMYB2* transcript levels strongly increased after IAA treatment, reaching their highest levels at 12 h after treatment. In contrast, a rapid increase in *HcMYB2* and *HcTPS5* expression was observed at 2 h after treatment (Figure 3C). Volatile biosynthesis genes (*HcTPS5* and *HcBSMT2*) were also upregulated at 12 h after IAA treatment (Figure 3D). The results suggest that the biosynthesis of floral volatiles is spatially and temporally regulated by HcMYB1 and HcMYB2, which are strongly associated with auxin-induced volatile emissions in *H. coronarium*.

**FIGURE 4:**
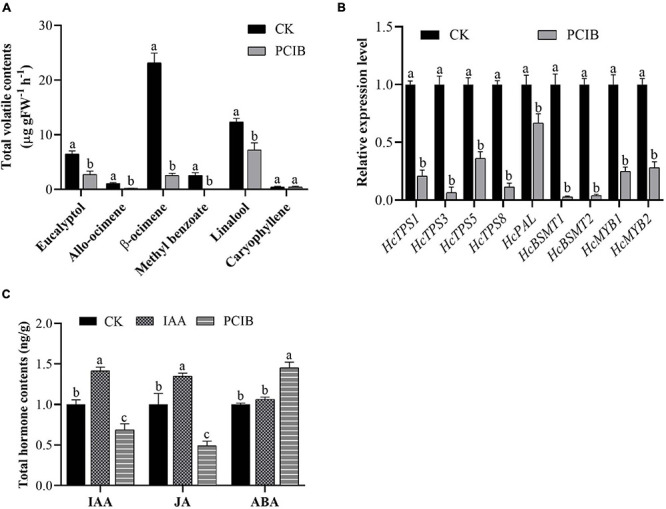
Marked decrease in scent emission, decreased expression level of key structural volatile synthesis genes, and total IAA, JA, and ABA contents under PCIB treatment. **(A)** Headspace analyses of individual scent compounds under PCIB treatment using GC–MS. **(B)** Relative expression level of key structural genes under PCIB treatment. GAPDH was used as an endogenous control. Bars represent the means ± SD (*n* = 3–5). **(C)** Total pool contents of IAA, JA, and ABA in response to auxin and PCIB treatment. Each time point represents the average of three to four independent experiments, with SD indicated by vertical lines. Graphs represent the average of three independent experiments. Different letters on bars indicate statistically significant differences (*P* < 0.05).

We also examined the effect of p-chlorophenoxyisobutyric acid (PCIB) (inhibit auxin action) on the floral volatile compounds (Figure 4). In contrast to auxin, the emission of floral volatile compounds decreases. Under PCIB treatment, the contents of eucalyptol, allo-ocimene, β-ocimene, methyl benzoate, and linalool were decreased by 57, 81, 89, 100, and 42%, respectively, compared to those in control flowers not treated with PCIB (Figure 4A). As expected, similar to IAA, the volatile contents of caryophyllene do not change significantly. We perform qRT-PCR analysis to characterize the expression level of key genes under PCIB treatment. The expression level of key volatile biosynthesis genes *HcTPS1*, *HcTPS3*, *HcTPS5*, *HcTPS8*, *HcPAL*, *HcBSMT1*, *HcBSMT2*, *HcMYB1*, and *HcMYB2* were downregulated by 79, 93, 64, 89, 33, 97, 96, 75, and 72%, respectively, relative to control (Figure 4B). The data endorse the aforementioned findings that auxin plays a crucial role in the biosynthesis of floral volatile compounds.

Furthermore, we quantify the total hormone contents under IAA and PCIB treatment using ultra-performance liquid chromatography-tandem mass spectrometer (UPLC-MS/MS). The data showed that under IAA treatment, the total IAA and jasmonic acid (JA) contents were increased by 42 and 35% compared to control. Likewise, the total IAA and JA contents were decreased by 31 and 51%, respectively, while abscisic acid (ABA) contents were increased by 45% under PCIB treatment (Figure 4C). However, the ABA contents do not change significantly under IAA treatment, suggesting that auxin might play a key role in the biosynthesis of floral volatile compounds via crosstalk with the abovementioned hormones.

### HcMYB1 and HcMYB2 Localize to the Nucleus and Exhibit Transactivation Activity

**FIGURE 5:**
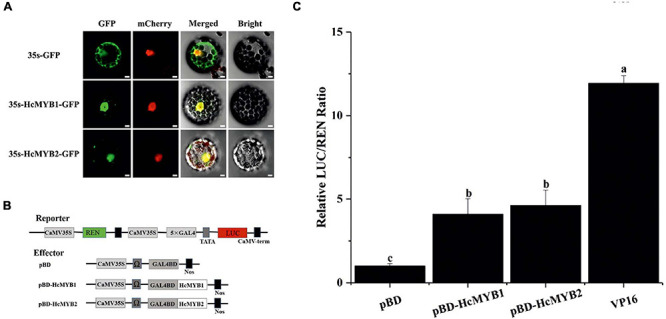
HcMYB1 and HcMYB2 show nuclear localization in *Arabidopsis* protoplasts. **(A)** The images were obtained using a confocal laser scanning microscope and processed in adobe Photoshop. GFP: green fluorescent protein. mCherry: nuclear marker. Merged: combined GFP and mCherry signals. Bright: bright field. Bars: 10 μm. **(B)** Diagram of the reporter and effector plasmids. **(C)** Transcriptional activity of HcMYB1 and HcMYB2 together with DNA via GAL4 BD (pBD-HcMYB1 and pBD-HcMYB2) transiently co-expressed in *N. benthamiana* cells. The activity is presented as the ratio of LUC to REN luminescence. The VP16 and pBD represent the positive and negative control, respectively. The ratio of LUC/REN of the empty pBD vector is used as the calibrator (set as 1). Data are represented as means ± standard deviation of three replicates. Compared with the pBD, significant differences at the level of *P* < 0.05 analyzed by the student’s *t*-test, are indicated by different letters above the bar.

Most MYB TFs specifically localize to the nucleus (Zou et al., 2008; Zhu et al., 2015; Zhou et al., 2017). However, some MYB TFs localize to both the nucleus and cytoplasm (Li et al., 2006a). The nuclear localization prediction server WoLF PSORT^1^ predicted that HcMYB1 and HcMYB2 localize to the nucleus. To assess this prediction, we generated HcMYB1-GFP and HcMYB2-GFP constructs in which these genes were driven by the CaMV *35S* promoter and used them to transform *Arabidopsis* protoplasts. In HcMYB1-GFP- and HcMYB2-GFP-transformed protoplasts, observed green fluorescent protein (GFP) signals specifically in the nuclei, whereas control (GFP) protoplasts showed a ubiquitous distribution of GFP throughout the protoplasts (Figure 5A). We included nuclear localization signal (NLS)-mCherry in each transformation as a marker for nuclear localization. These results demonstrate that HcMYB1 and HcMYB2 are nucleus-localized proteins, which is in keeping with their expected roles as transcription factors.

Transcription factors regulate their target genes via transactivation activity. To investigate the transactivation activities of HcMYB1 and HcMYB2, we performed transient expression analysis in *Nicotiana benthamiana* leaves. We fused five copies of the GAL4 DNA-binding element (GAL4BD) and the minimal TATA region (5′-TATAAA-3′) of the *35S* promoter to the firefly luciferase (*LUC*) reporter; the Renilla luciferase (*REN*) reporter gene driven by the *35S* promoter as the reporter vector. The LUC/REN ratio from the reporter vector was used as an internal control. We constructed effector plasmids harboring the ORFs of *HcMYB1* and *HcMYB2* (Figure 5B). Unlike the GAL4BD negative control (empty vector, pBD), HcMYB1 and HcMYB2 activated the *LUC* reporter gene. The LUC/REN ratios of HcMYB1, HcMYB2, and GAL4BD-VP16 were 3. 1-, 3. 7-, and 11.1-fold higher, respectively, compared to the negative control (Figure 5C). These results indicate that HcMYB1 and HcMYB2 function as transcriptional activators.

### Virus-Induced Gene Silencing of *HcMYB1* and *HcMYB2* in Flowers Modifies the Emission Levels of Volatiles

**FIGURE 6:**
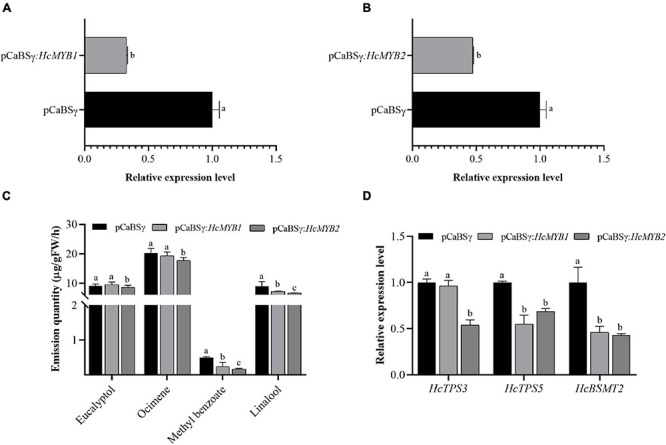
*HcMYB1* and *HcMYB2* gene silencing alter *HcTPS3*, *HcTPS5*, and *HcBSMT2* expression and volatile emission in flowers. **(A,B)** Relative expression levels are presented as fold change values. **(C)** Emission levels of floral volatiles. **(D)** Expression levels of *HcTPS3*, *HcTPS5*, and *HcBSMT2*. Data represent the mean ± SD (*n* = 3–5). Different letters indicate significant differences (*P* < 0.05).

To investigate the potential involvement of *HcMYB1* and *HcMYB2* in floral scent formation, we suppressed their expression through virus-induced gene silencing (VIGS) in flowers (Renner et al., 2009; Yuan et al., 2011). We confirmed that this led to significant decreases in *HcMYB1* and *HcMYB2* transcript levels compared to those in unsilenced control flowers (Figures 6A,B). The contents of the volatiles methyl benzoate and linalool in flowers decreased by approximately 57 and 21%, respectively, in response to *HcMYB1* silencing, whereas the eucalyptol and ocimene contents did not change significantly (Figure 6C). In *HcMYB2*-silenced flowers, the contents of methyl benzoate, linalool, ocimene, and eucalyptol decreased by 68, 37, 18, and 17%, respectively, compared to the control (Figure 6C). We also analyzed the expression levels of key volatile biosynthesis genes (*HcTPS3*, *HcTPS5*, and *HcBSMT2*) in *H. coronarium*. In *HcMYB1*-silenced flowers, *HcTPS5* and *HcBSMT2* were significantly downregulated, whereas *HcTPS3* did not exhibit any significant changes in expression, compared to the control. Furthermore, in *HcMYB2*-silenced flowers, *HcTPS3*, *HcTPS5*, and *HcBSMT2* were all significantly downregulated compared to the control (Figure 6D). These results indicate that HcMYB1 and HcMYB2 play important and overlapping roles in the formation of floral volatiles in *H. coronarium*.

### HcMYB1 and HcMYB2 Activate Structural Genes Involved in the Volatile Biosynthetic Pathway

MYB TFs transcriptionally regulate several genes by binding to the MEB [(T)(T)TGAC(C/T)] sequences in their promoters (Rushton et al., 2010). *In silico cis*-element analysis revealed the presence of MYB-binding motifs in *HcBSMTs* and *HcTPSs* sequences. MYB-core binding motifs were present in ten out of twelve *HcBSMTs*. The number of MYB-binding motifs varies from one to thirteen. Interestingly, the number of MYB-bindings motifs in *HcBSMT2* was highest compared to other *HcBSMTs* (Supplementary Table 1). Similarly, MYB-core binding motifs were found in sixty out of sixty-two *HcTPSs*. The promoter sequence analysis of *HcBSMT2* (1131 bp) and *HcTPS5* (1555 bp) revealed the presence of MYB-core binding motifs in their sequences. There were 13 and 5 copies of MYB-binding motifs in the sequences of *HcBSMT2* and *HcTPS5*, respectively, suggesting that HcMYB1 and HcMYB2 might target these genes.

**FIGURE 7:**
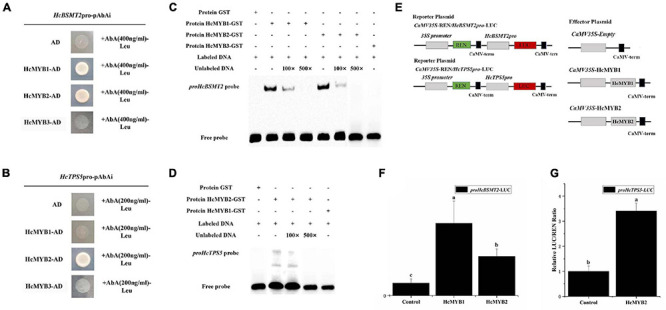
HcMYB1 and HcMYB2 interact with the *HcBSMT2* and *HcTPS5* promoters. **(A)** Interactions of HcMYB1 and HcMYB2 with the *HcBSMT2* promoter. **(B)** Interactions of HcMYB1 and HcMYB2 with the *HcTPS5* promoter. HcMYB3 and empty vector were used as a control. **(C)** Binding activity of HcMYB1 and HcMYB2 with the *proHcBSMT2* probe. **(D)** Binding activity of HcMYB2 with the *proHcTPS5* probe. **(E)** Diagram of the reporter and effector plasmids. **(F)** HcMYB1 and HcMYB2 transactivate the *HcBSMT2* promoter. **(G)** HcMYB2 transactivates the *HcTPS5* promoter. The promoter activity is presented as the ratio of LUC to REN luminescence. Different letters on bars indicate statistically significant differences (*P* < 0.05).

To determine whether HcMYB1 and/or HcMYB2 bind to the promoters of *HcBSMT2* and *HcTPS5*, we performed a yeast one-hybrid (Y1H) assay. Bait strains co-expressing HcMYB1 and HcMYB2 and harboring *proHcBSMT2* grew well in SD-Leu medium containing the antibiotic aureobasidin A (AbA), whereas bait strains harboring *proHcTPS5* grew well only when they expressed HcMYB2 (Figures 7A,B). These results indicate that HcMYB1 and HcMYB2 bind to the *HcBSMT2* promoter, while HcMYB2 binds to the *HcTPS5* promoter.

To confirm the binding ability of HcMYB1 and HcMYB2 to the *HcBSMT2* and *HcTPS5* promoters, we performed an electrophoretic mobility shift assay (EMSA) using GST-HcMYB1 and GST-HcMYB2 in *Escherichia coli*. The probes used for *proHcTPS5* and *proHcBSMT2* were 49 bp, which start from (+)1168 to (+)1216 and (+)291 to (+)339, respectively. The sequences of the probes are listed in Supplementary Table 2. Purified recombinant GST-HcMYB1 and GST-HcMYB2 fusion proteins bound to biotin-labeled probes derived from the *HcBSMT2* promoter, leading to a mobility shift, whereas no mobility shift occurred in the presence of GST alone (Figure 7C). Next, we performed a competition assay, which showed that adding a 100-fold amount of unlabeled probe molecules (as compared to the labeled molecules) to the binding reaction reduced the intensity of the protein-DNA complex signal, and adding 500-fold unlabeled probes prevented any protein-DNA complex from being detected (Figure 7C). We also observed binding between HcMYB2 and a biotin-labeled probe from the *HcTPS5* promoter (Figure 7D). These results endorse the aforementioned data that HcMYB1 binds to the *HcBSMT2* promoter and HcMYB2 binds to the *HcBSMT2* and *HcTPS5* promoters.

To test the ability of HcMYB1 and HcMYB2 to activate the *HcBSMT2* and *HcTPS5* promoters, we performed a dual-luciferase assay. We individually cloned the promoter regions of *HcBSMT2* and *HcTPS5* into reporter plasmids and the ORFs of *HcMYB1* and *HcMYB2* into effector plasmids (Figure 7E). HcMYB1 and HcMYB2 significantly enhanced *HcBSMT2* promoter activity (by 4.8-fold and 2.2-fold, respectively) compared to the control (Figure 7D). Meanwhile, HcMYB2 significantly enhanced *HcTPS5* promoter activity (by 2.4-fold) compared to the control (Figure 7F). Therefore, HcMYB1 and HcMYB2 activate the *HcBSMT2* promoter and HcMYB2 activates the *HcTPS5* promoter, indicating that these TFs have different target genes in *N. benthamiana* leaves. These findings indicate that both HcMYB1 and HcMYB2 are transcriptional activators of volatile biosynthesis genes in flowers.

### HcMYB1 Interacts With the Auxin-Responsive Protein HcIAA4 by Y2H and BiFC Assays

**FIGURE 8:**
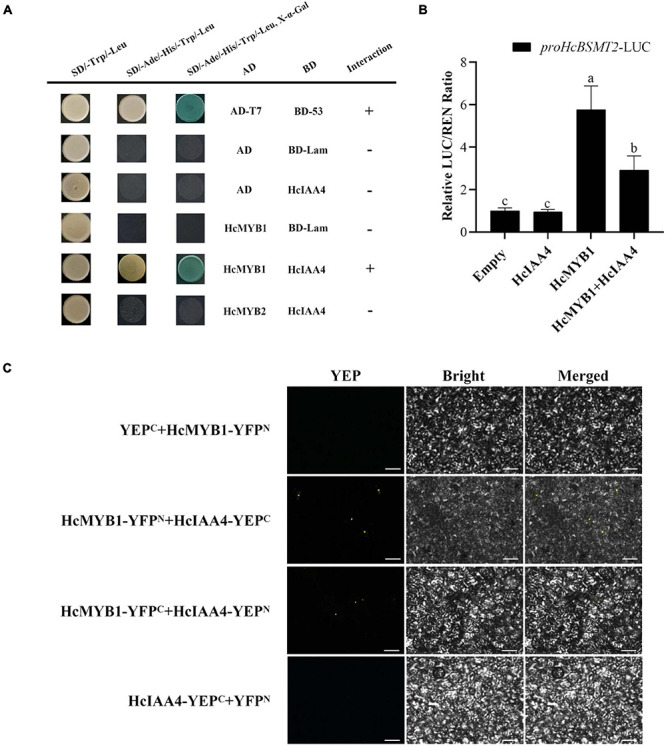
HcMYB1 interacts with HcIAA4. **(A)** Analysis of the interactions of HcIAA4, HcMYB1, and HcMYB2 via yeast two-hybrid assay. Yeast cells were transformed with DBD–HcARF4 + AD–HcMYB1, DBD–HcARF4 + AD–HcMYB2, DBD + AD–HcMYB1, DBD–HcIAA4 + pGADT7-T, pGBKT7-53 + pGADT7-T, DBD–HcIAA4 + pGADT7-T or pGBKT7-Lamin + pGADT7-T. The vectors were transformed into yeast strain Y2HGold and then transformants were screened by plating on SD/-Trp/-His/-Ade + X-α-gal medium. BD-Lam was used as a negative control. ADT7-BD53 was used as a positive control. Three independent replicates were performed. **(B)** BiFC assay between HcMYB1 and HcIAA4 in *N. benthamiana* leaves. Fusion constructs YFP^C^ + HcMYB1-YFP^N^, HcMYB1-YFP^N^ + HcIAA4-YFP^C^, HcMYB1-YFP^C^ + HcIAA4-YFP^N^, and HcIAA4-YFP^C^ + YFP^N^ were co-infiltrated into *N. benthamiana* leaves and observed for fluorescence complementation. Bars, 5 μm. **(C)** The activation ability of HcMYB1 is modulated by HcIAA4. Different letters on bars indicate statistically significant differences (*P* < 0.05).

MYB proteins interact with many other proteins involved in hormone signal transduction, such as the jasmonic acid (JA)-responsive repressor proteins of the JASMONATE ZIM-DOMAIN (JAZ) family and the ABA signal receptor protein PYRABACTIN RESISTANCE LIKE (PYL) (Qi et al., 2014; Zhao et al., 2014). In a Y2H assay, HcMYB1 interacted with the auxin-responsive protein HcIAA4, whereas HcMYB2 did not (Figure 8A). To verify the interaction between HcMYB1 and HcIAA4, we performed a BiFC assay. Expressing the N-terminal half of YFP fused to HcMYB1 (HcMYB1-YFP^N^) and the C-terminal half of YFP fused to HcIAA4 (HcIAA4-YFP^C^) in *N. benthamiana* leaves resulted in fluorescence. Moreover, the reciprocal experiment with the C-terminal half of YFP fused to HcMYB1 (HcMYB1-YFP^C^) and the N-terminal half of YFP fused to HcIAA4 (HcIAA4-YFP^N^) also resulted in fluorescence and the control combinations of YFP^C^ + HcMYB1-YFP^N^ and HcIAA4-YFP^C^ + YFP^N^ did not result in fluorescence (Figure 8B). To elucidate the functional significance of the interaction between HcMYB1 and HcIAA4, we co-transformed *N. benthamiana* leaves with the same amounts of effectors carrying HcMYB1 and/or HcIAA4 in combination with the *HcBSMT2pro*-LUC reporter constructs. The effect of HcMYB1 on *HcBSMT2pro* expression was repressed in the presence of HcIAA4 (Figure 8C). These results demonstrate that HcMYB1 directly activates *HcBSMT2* expression, which is modulated by its interacting partner HcIAA4.

## Discussion

The transcriptional regulatory network governing floral scent emission has not been thoroughly elucidated. To date, only a few TFs that regulate the expression of scent-related genes have been identified (Katiyar et al., 2012; Abbas et al., 2021b). R2R3-MYB TFs are key regulators of the phenylpropanoid and terpenoids biosynthetic pathway in plants (Du et al., 2009; Zhu et al., 2015; Yang et al., 2020). MYB TFs in the same subgroup have similar functions (Zhu et al., 2015). Here, we used previously characterized R2R3-MYB proteins involved in secondary metabolism to construct a phylogenetic tree with HcMYB1 and HcMYB2 from *H. coronarium* (Figure 1B). HcMYB1 was clustered in Group III with AtMYB77, which modulates auxin signal transduction (Shin et al., 2007), and AtMYB44, a stress-responsive protein involved in senescence and ABA signaling (Jaradat et al., 2013), suggesting that HcMYB1 might play a key role in hormone signaling. Meanwhile, HcMYB2

was classified into Group I along with AmMYB305, AmMYB340, AtMYB24, AtMYB21, PhEOBII, and FaEOBII (Figure 1). These R2R3-MYB TFs regulate the metabolic pathway of the volatile compound phenylpropanoid (Uimari and Strommer, 1997; Shin et al., 2002; Li et al., 2006b; Liu et al., 2009; Spitzer-Rimon et al., 2010; Medina-Puche et al., 2015), suggesting that HcMYB2 might be involved in regulating the floral volatile metabolic pathway in *H. coronarium*.

### *HcMYB1* and *HcMYB2* Are Expressed During Specific Stages of Floral Development and Are Correlated With Volatile Production

The production and emission of fragrance compounds by flowers are strictly regulated during the floral lifespan and often peak when the flower is in full bloom and pollinators are active (Dudareva et al., 2013; Muhlemann et al., 2014; Abbas et al., 2017). Methyl benzoate and linalool are the main phenylpropanoids and terpenoid volatiles that contribute to flower scent in *H. coronarium* (Fan et al., 2003, 2007; Báez et al., 2011; Yue et al., 2015; Abbas et al., 2021a). We observed that the expression of *HcMYB1* changed during flower development, with the highest expression level detected at the full bloom stage (F5) (Figure 2B. A similar expression pattern was detected in lilac (*Syringa oblata*) during different stages of flower development, as two R2R3-MYB TF genes were upregulated at the full-bloom stage compared to the bud stage (Zhu et al., 2015). Similarly, the expression level of *FhMYB5* gradually increased during the flower developmental stages, resembling anthocyanin biosynthesis pattern and function in the flavonoid pathway in *Freesia hybrida* (*F. hybrida*) (Li et al., 2019). In *Rosa hybrida*, mRNA levels of a putative scent-related gene (*RhMYB1*) were developmentally regulated peaking at full bloom stage similar with other rose scent-related genes, such as phenylacetaldehyde synthase *RhPAAS*, the sesquiterpene synthase *RhGDS*, the alcohol acetyltransferase *RhAAT* and the orcinol O-methyltransferases (*OOMT*) (Lavid et al., 2002; Yan et al., 2011).

Interestingly, the expression patterns of *HcMYB1* and *HcMYB2* during development were similar to those of *HcBSMT2* and *HcTPS5*, which are responsible for the formation of the volatiles methyl benzoate and linalool, respectively (Figure 2D). Both compounds reach their highest levels in flowers at the full bloom stage (Yue et al., 2015). Similarly, *HcBSMT2* and *HcTPS5* expression levels were highest in flowers during the periods when the largest amounts of volatiles were released (Supplementary Figure 5). Thus, the expression of TF genes (*HcMYB1* and *HcMYB2*) and structural genes (*HcBSMT2* and *HcTPS5*) was associated with flower development and the production of high levels of volatiles (Supplementary Figure 1). In *F. hybrida* and *A. thaliana*, FhMYB21L1 and FhMYB21L2, TF genes were synchronously expressed with *FhTPS1* and could activate its expression significantly (Yang et al., 2020). These results suggest that HcMYB1 and HcMYB2 regulate volatile production during flower development, which is similar to the roles of R2R3-MYBs PhEOBII and FaEOBII in petunia and strawberry, respectively (Van Moerkercke et al., 2011; Medina-Puche et al., 2015).

### *HcMYB1* and *HcMYB2* Expression Is Regulated by Auxin Like That of Other Scent-Related Genes in *H. coronarium*

The volatile biosynthesis pathway, and particularly the emission of methyl benzoate and linalool, is induced by IAA, suggesting that IAA regulates the expression of transcription factors or key enzymes involved in this pathway at the protein or transcript level. In the current study, we demonstrated that auxin induces the expression of both TF genes (*HcMYB1* and *HcMYB2*) and key biosynthesis genes [phenylalanine ammonia lyase (*PAL*), *BSMT*, and *TPS*] (Figures 3B,C). IAA treatment also upregulated *HcBSMT2* and *HcTPS5* expression, especially at 12 h (Figure 3D). In *A. thaliana*, expression of *AtTPS21* and *AtTPS11* was induced by the phytohormones, and both inductions require AtMYC2 (Hong et al., 2012). R2R3-MYBs such as *AtMYB77* and *AtMYB44* are involved in the response to auxin signaling in *Arabidopsis* (Shin et al., 2007; Yamaguchi et al., 2013). Similarly, *FaMYB10* and *FaEOBII* are regulated by auxin in strawberry (Perkins-Veazie, 1995; Chai et al., 2011; Jia et al., 2011). On the other hand, auxin contents decrease and *FaMYB10* and *FaEOBII* expression increases during fruit development in strawberry (Medina-Puche et al., 2014, 2015). During flower development in *H. coronarium*, auxin contents increased and *HcMYB1* and *HcMYB2* expression increased (Figures 2C,E). The differences in auxin response patterns between the *HcMYB* and *FaMYB* genes may be due to the evolutionary distance between *H. coronarium* (Zingiberaceae) and *F.* × *ananassa* (Rosacea). To validate the function of auxin, the flowers were treated with PCIB which is widely used to inhibit auxin action (Oono et al., 2003). The data showed that in contrast to auxin, the emission of main floral volatiles and expression level of aforementioned key structural volatile synthesis genes significantly downregulated (Figures 4A,B). The following data endorse the abovementioned findings that auxin plays an essential role in floral scents. Furthermore, relative to the control, total IAA and JA contents significantly upregulated and downregulated under auxin and PCIB treatment, respectively (Figure 4C). Several studies showed that MYB TF respond to various phytohormones. Under JA treatment, the transcript abundance of *Pinus taeda PtMYB14* and *PtMYB13* rapidly increased by 14-fold and 2-fold, respectively, while *Picea glauca PgMYB14* and *PgMYB15* transcripts increased 4-fold and 2-fold. Furthermore, the characterization of the aforementioned TF genes reveals *PtMYB14* as a putative regulator of an isoprenoid and flavonoid-oriented response in conifers (Bedon et al., 2010). In Apples, MdMYB9 and MdMYB11 were involved in the regulation of the JA-induced biosynthesis of anthocyanin and proanthocyanidin (An et al., 2014). The regulatory patterns of *MYBs* are dependent on developmental stage, tissue type, and environmental conditions. Much remains to be learned about the mechanistic basis of the responses of MYB TFs to auxin signaling molecules during volatile formation.

### HcMYB1 and HcMYB2 Activate Key Structural Genes Involved in Volatile Biosynthesis in *H. coronarium* Flowers

The structural genes *HcBSMT2* and *HcTPS5* are essential for the formation of methyl benzoate and linalool, respectively, in *H. coronarium* flowers (Yue et al., 2015). Notably, we detected MYB-binding elements in the promoters of *HcBSMT2* and *HcTPS5* (Supplementary Table 1). This result is supported by the finding that HcMYB1 transactivates the *HcBSMT2* promoter and that HcMYB2 transactivates the *HcBSMT2* and *HcTPS5* promoters (Figure 7). In certain plants, floral scent biosynthesis is dependent on transcriptional regulation, and TFs control volatile emissions (Colquhoun et al., 2011; Muhlemann et al., 2012). ODO1 was the first R2R3-type MYB transcription factor shown to regulate the benzenoid biosynthesis pathway in petunia, followed by the R2R3-MYB TFs EOBI and EOBII. ODO1 strongly influences the floral scent pathway by regulating the transcript levels of many key genes (*PAL*, *CM*, *DAHPS*, *SAMS*, and *EPSPS*). Meanwhile, *ODO1* is directly regulated by EOBII. Moreover, EOBI directly binds to and activates the promoters of *ODO1*, *IGS*, and *PAL* to regulate scent production (Verdonk et al., 2005; Spitzer-Rimon et al., 2010, 2012). In *F. hybrida*, FhMYB5 and FhbHLH mainly contribute to the regulation of anthocyanin and proanthocyanidin via activating the expression of biosynthetic genes (*FhCHS*, *FhCHI*, *FhF3H*, *FhF3′H*, *FhF3′5′H*, and *FhDFR*) involved in the flavonoid pathway (Li et al., 2019). In spearmint, MsMYB negatively regulates monoterpene production and suppresses the expression of geranyl diphosphate synthase (Reddy et al., 2017). Likewise, several R2R3-MYB transcription factor have been identified which are potentially involved in the regulation of flavonoid biosynthesis via controlling the expression of structural genes (Cao et al., 2021; Zhang et al., 2021). Similarly, *Lilium* hybrid *ODO1* (*LhODO1*) regulates fragrance biosynthesis via regulating the expression of structural genes involved in the shikimate and benzenoid/phenylpropanoid pathway (Yoshida et al., 2018).

In *H. coronarium*, HcMYB1 and HcMYB2 directly activate the methyl benzoate biosynthesis gene *HcBSMT2*, whereas HcMYB2 activates the linalool biosynthesis gene *HcTPS5*. Therefore, HcMYB2 activates two different groups of volatile biosynthesis genes, suggesting it plays a dual role in controlling both the phenylpropanoid and terpenoid pathways. Several R2R3-MYB TFs regulate the biosynthesis of one or more units of phenylpropanoid-derived compounds, such as MdMYB3, AtMYB4, and AtMYB12 (Aharoni et al., 2001; Deluc et al., 2006, 2008; Luo et al., 2008; Rommens et al., 2008; Liu J. et al., 2015). Here, we examined the effects of HcMYB1 and HcMYB2 on floral scent via gene silencing (Figure 6). Linalool and methyl benzoate levels significantly decreased in flowers when *HcMYB1* or *HcMYB2* was silenced, confirming the direct connection between the functional activity of these two TFs and volatile biosynthesis. The silencing of *HcMYB1* or *HcMYB2* also led to the downregulation of key structural scent-related genes (*HcTPS3*, *HcTPS5*, and *HcBSMT2*) from the terpenoid and phenylpropanoid pathways (Figure 6D). Similar results were obtained in petunia, where the silencing of R2R3-MYB (*ODO1*) led to the downregulation of several scent-related genes (Spitzer-Rimon et al., 2012). In addition, overexpressing *PAP1* from *Arabidopsis* modulated the accumulation of terpenoid and phenylpropanoid scent compounds in rose flowers (Zvi et al., 2012). Nevertheless, before this study, little was known about the transcriptional regulatory mechanism underlying scent compound biosynthesis in non-model fragrance plants such as *H. coronarium*.

### HcIAA4 Interacts With and Modulates the Transcriptional Activity of HcMYB1

MYB TFs form complexes by interacting with other proteins, such as MYB-helix-loop-helix (bHLH)-WD40 proteins involved in regulating anthocyanin biosynthesis (Zhou and Memelink, 2016). MYB TFs also interact with other proteins involved in hormone signaling pathways, such as JAZ and PYL, which are crucial components of the JA and ABA signal-transduction pathways, respectively (Qi et al., 2014; Zhao et al., 2014). However, little is known about the interactions of MYB TFs with proteins in the auxin-signaling pathway. In *Arabidopsis*, the auxin signaling pathway repressor Aux/IAA (AtIAA29) interacts with the TF WRKY57 to mediate leaf senescence (Jiang et al., 2014). It was also observed that both HcMYB1 and HcIAA4 showed high protein expression in flowers (Supplementary Figure 4). In the current study, we uncovered an interaction between HcMYB1 and the auxin-responsive protein HcIAA4 via Y2H and BiFC assays (Figures 8A,B). We also demonstrated that HcIAA4 represses the activity of HcMYB1 (Figure 7C). Similarly, in *Arabidopsis*, AtJAZ proteins interact with MYBs such as MYB75, thereby decreasing their transcriptional activity (Qi et al., 2014). In *A. thaliana* and *F. hybrida*, MYB21 interacts with MYC2 to form MYB–bHLH complex to regulate the expression of TPS genes and floral scent emission in flowers (Yang et al., 2020). In *Fagopyrum tataricum*, the repressive activities of FtMYBs are directly enhanced by their interactions with FtSAD2 or FtJAZ1 (Zhang et al., 2018). The identification of protein–protein interactions between MYB TFs and other proteins provides clues about the regulation of gene expression and secondary metabolism during volatile biosynthesis.

In petunia, a network comprising three R2R3 MYB TFs (EOBI, EOBII, and ODO1) regulates flower-specific genes in the phenylpropanoid volatile biosynthesis pathway (Verdonk et al., 2005; Spitzer-Rimon et al., 2010, 2012; Van Moerkercke et al., 2011). Similarly, FaMYB10 regulates *FaEOBII* expression in strawberry (Medina-Puche et al., 2014, 2015). In *A. thaliana*, MYC2 interacts with DELLA protein to regulate the expression of sesquiterpene synthase genes (*TPS21* and *TPS11*), and the expression of *TPS21* and *TPS11* was modulated by phytohormones (Hong et al., 2012). Likewise, LcMYB1 interacts with LcbHLH to regulate the expression of key structural anthocyanin biosynthesis genes in *Litchi chinensis* (Lai et al., 2016). The roles of MYB proteins in scented ornamental plants uncovered in the current study sheds light on the evolution of this important transcription factor family, providing new insights into how they regulate the biosynthesis of secondary metabolic compounds, including terpenoids and phenylpropanoids, in plants. The key role of MYB TFs in controlling the biosynthesis of volatile compounds highlights the potential of engineering these TFs to enhance the economic value of ornamental plant species.

In conclusion, we demonstrated that the IAA-responsive, flower-specific R2R3-MYB TFs HcMYB1 and HcMYB2 function as activators of terpenoid and phenylpropanoid biosynthesis in *H. coronarium*. Both HcMYB1 and HcMYB2 interact with the promoter of *HcBSMT2*, encoding the key enzyme for methyl benzoate biosynthesis. Furthermore, HcMYB2 regulates the expression of *HcTPS5*, which plays a key role in linalool biosynthesis. Finally, we showed that auxin takes part in volatile biosynthesis by regulating the expression of R2R3-HcMYB transcription factor genes via protein–protein interactions in *H. coronarium*. Our findings provide important insights into the roles of auxin and MYB TFs in the biosynthesis of floral scent compounds, laying the foundation for plant metabolic engineering efforts.

## Materials and Methods

### Plant Material and Hormone Treatment

*Hedychium coronarium* plants were grown at South China Agricultural University under natural light conditions. For RNA extraction, the plant materials were harvested, immediately frozen in liquid nitrogen, and stored at −80°C. To analyze tissue-specific gene expression patterns, three tissues were used: fully open flowers, mature green leaves, and healthy rhizomes of 2-year-old *H. coronarium* plants. The process of flower development was divided into six stages: tight green bud stage (F1), white bud stage (F2), initial flowering stage (F3), half-open stage (F4), full-bloom stage (F5), and flower senescence (F6).

For IAA treatment, flowers at the F2 stage were cut into 35-cm pieces, placed in sterile water containing 100 μM IAA, and incubated for 12 h in a growth chamber under a 14/10 h light/dark cycle at 25°C. IAA and PCIB stock solution was prepared as per the manufacturer’s protocol. Briefly, 18.79 mg IAA powder was dissolved in 1.5 mL methanol and diluted in 100 mL sterilized water. Likewise, 321 mg PCIB powder was dissolved and diluted as aforementioned conditions. Thereafter, the detached flowers were placed in a flask containing 100 mL solution and covered with a silver sheet to protect them from degradation. The control flowers were kept in with a similar amount of volume of sterile water without IAA under the same aforementioned conditions. Volatile content was analyzed in flowers at the full-bloom stage. After analysis, all samples were frozen in liquid nitrogen and stored at −80°C for further experiments. Three to five independent experiments was performed with each experimental variant. *A. thaliana* and *N. benthamiana* plants used for subcellular localization and BiFC assays were grown in a growth chamber at 24°C under a 12/12 light/dark cycle.

### Sequence Alignment and Phylogenic Analysis

The sequences of *HcMYB1* and *HcMYB2* were obtained from a flower RNA-seq database for *H. coronarium* (SRP049915). The related protein sequences were retrieved from the NCBI database^2^. The protein sequences were aligned, and a phylogenetic tree based on the R2R3-MYB domain was constructed using Clustal Ω (Sievers et al., 2011) and MEGA X (Kumar et al., 2018) software.

### RNA Isolation, cDNA Synthesis, and qRT-PCR

Total RNA was isolated from flowers at different stages of development and different organs using a HiPure Plant RNA Mini Kit (Magen) following the manufacturer’s protocol. Each 1 μg RNA sample was reverse transcribed using a PrimeScript RT Reagent Kit with Genomic DNA Eraser (Takara) following the manufacturer’s suggestions. To generate primers for reverse-transcription PCR (RT-PCR), the specific sequences of genes in *H. coronarium* were selected, and primers for the genes were designed with Primer 5.0 software (Abbas et al., 2020). The reaction mixtures (20 μl) included 10 μL pink SYBR mix, 7.2 μl distilled water, 0.4 μl each primer (10 μM), and 2.0 μl template cDNA. The expression values were calculated using the 2^–ΔΔCt^ method (Livak and Schmittgen, 2001). A similar procedure was performed for all treatments using specific primers listed in Supplementary Table 2.

### Subcellular Localization Assay

The full-length fragments excluding the stop codon were fused with the *GFP* gene in the p35S-EGFP-1 vector using *Sma*I at the 5′ end and *Spe*I at the 3′ end. The isolation and transformation of *Arabidopsis* protoplasts were performed as described by Yoo et al. (2007). The protoplasts were observed and photographed at 18 h after transformation under a confocal laser-scanning microscope.

### Virus-Induced Gene Silencing

Barley stripe mosaic virus (BSMV) was used for VIGS, as this system has successfully been used in monocots (Renner et al., 2009; Yuan et al., 2011). The pCaBSγ vector was linearized with *Apa*I before inserting the fragments. To specifically silence *HcMYB1* and *HcMYB2*, a 280-bp fragment of each gene (from the 3′ end) was amplified by PCR from *H. coronarium* cDNA and inserted into pCaBSγ to produce pCaBSγ:*HcMYB1* and pCaBSγ:*HcMYB2*, respectively. The cultures were harvested by centrifugation at 5000 rpm for 10 min and resuspended in infiltration buffer (10 mM MgCl_2_, 0.1 mM acetosyringone, 10 mM MES, pH 5.6) to a final OD_600_ of ∼1. For VIGS, flowers at the F1 stage were collected, dipped in the bacterial suspension, and vacuum infiltrated at 0.8 MPa. After the vacuum was released, the flowers were washed in deionized water, placed into liquid MS medium, and cultured under a 12/12 h light/dark cycle at 16°C for 5 days. Total floral volatile compounds were analyzed at the full-bloom stage via gas chromatography-mass spectrometer (GC-MS); the experiment was replicated three to four times.

### Yeast One-Hybrid Assay

The yeast-one-hybrid assay was performed using the Gold Yeast One Hybrid System (Clontech, Takara). To generate bait-specific reporter strains, a 1131-bp fragment (−1 to −1131 bp upstream of ATG) of the *HcBSMT2* promoter and a 1555-bp fragment (−1 to −1555 bp upstream of ATG) of the *HcTPS5* promoter were inserted into pAbAi to generate *HcBSMT2*-pAbAi and *HcTPS5*-pAbAi, respectively. The plasmids were integrated into the genome of yeast strain Y1H Gold (Clontech, Takara) via homologous recombination, and transformed colonies were selected on uracil-deficient synthetic dropout (SD/-Ura) medium. Different concentrations of the antibiotic AbA were used to select the bait strains. To generate the prey constructs, full-length *HcMYB1* and *HcMYB2* were cloned into pGAL4. The prey constructs were transformed into yeast cells harboring the bait constructs. The *in vivo* DNA-binding activity was determined based on the growth status of the transformed yeast cells on leucine-deficient synthetic dropout (SD/-Leu) medium supplemented with the selected concentration of AbA after 3–5 days of cultivation at 30°C.

### Yeast Two-Hybrid Assay

The full-length *HcIAA2* and *HcIAA4* sequences were ligated into prey vector pGADT7 (AD). The coding sequences of *HcIAA4*, *HcMYB1*, and *HcMYB2* were cloned into bait vector pGBKT7 (BD). The prey construct and specific bait construct were co-transformed into yeast strain Y2HGold, which harbors four reporter genes (*Ade*, *MEL1*, *His*, and *AUR1*) under the control of a GAL4-responsive promoter. The empty pGADT7 vector was used as a blank control. The positive transformants that grew on SD/-Trp medium were inoculated onto SD plates (SD/-Leu-Trp-His-Ade) and incubated for 3–5 days at 30°C; transactivation activity was confirmed by growth on these plates. Yeast colonies expressing the α-galactosidase MEL1 turned blue upon the addition of X-α-Gal substrate (Clontech, TaKaRa). Primers used to amplify the genes are listed in Supplementary Table 2.

### Dual-Luciferase Transient Expression Assay

To assay the transcriptional activities of *HcMYB1* and *HcMYB2*, the full-length coding regions were independently fused to the GAL4 DNA-binding domain ligated to the pBD vector and used as the effectors. The reporter vector was modified from the pGreenII 0800-LUC vector, which includes the firefly luciferase gene (*LUC*) driven by the CaMV *35S* minimal promoter with five repeats of upstream activating sequence, as well as the *35S*-promoter-driven *Renilla reniformis* luciferase gene (*REN*) as an internal control. To examine the binding of HcMYB1 and HcMYB2 to the promoters of *HcBSMT2* and *HcTPS5*, the promoters were cloned into the pGreenII 0800-LUC double-reporter vector (Hellens et al., 2005), whereas *HcMYB1* and *HcMYB2* were cloned into the pGreenII 62-SK vector as effectors. The effector and reporter plasmids were electroporated into *Agrobacterium tumefaciens* strain EHA105 and injected into *N. benthamiana* leaves with a needleless syringe. After 3–5 days, leaves were collected and LUC and REN activities examined via a dual-luciferase assay (Promega, United States) using a Luminoskan Ascent Microplate Luminometer (Thermo Fisher, United States) following the manufacturer’s protocol. The transactivation ability and the binding activity of HcMYB1 and HcMYB2 are indicated by the ratio of LUC to REN. Four or five measurements were carried out for each combination and three independent experiments were performed.

### Electrophoretic Mobility Shift Assay

For EMSA, pGEX-4T-1 (GE Healthcare) was used to generate the GST-HcMYB1 and GST-HcMYB2 expression vectors, which were transformed into *E. coli* strain *BM* Rosetta (DE3). The expression of the recombinant fusion proteins was induced by adding 0.5 mM isopropyl-β-D-thiogalactopyranoside (IPTG). Following incubation at 28°C for 8 h, the fusion proteins were purified using Glutathione Superflow Resin (Clontech) according to the manufacturer’s instructions. The fragments (∼50 bp) containing putative MBE-binding sequences in the *HcTPS5* and *HcBSMT2* promoters were labeled with biotin. EMSA was carried out using a Light Shift Chemiluminescent EMSA Kit (Thermo Scientific) as previously described (Tan et al., 2019). The purified fusion protein was incubated with biotin-labeled DNA fragments and a 100-fold molar excess of unlabeled DNA fragments with the same sequences that were used as competitors; GST protein with labeled DNA was used as a negative control. The protein-DNA complexes were separated by 5% native polyacrylamide gel electrophoresis, detected based on chemiluminescence on a ChemiDoc MP Imaging System (Bio-Rad), and transferred onto a nylon membrane.

### BiFC Assays

The full-length *HcMYB1* and *HcIAA4* sequences were separately inserted into PUC-SPYNE and PUC-SPYCE to form HcMYB1-YFP^N^ and HcIAA4-YFP^C^, respectively. The empty vector (control) and recombinant plasmids were transformed into EHA105 competent cells, and different combinations of *Agrobacterium* cultures were co-infiltrated into *N. benthamiana* leaves. The plants were cultivated in an incubator under a 16 h/8 h light/dark cycle for 3 days, and the infiltrated leaves were visualized under a Leica DM RXA2 upright fluorescence microscope as described previously (Ke et al., 2019).

### Measuring IAA Contents in *H. corium* Flowers

The flower samples were ground to a fine powder in liquid nitrogen (N_2_) and transferred to 15 mL tubes containing 5 mL extraction solvent (2:1:0.002 [v/v/v] 2-propanol:H_2_O:HCl). The samples were sonicated for 15 min and incubated at 4°C for 30 min with shaking (100 rpm). After adding 5 mL dichloromethane, the samples were incubated under the same conditions and centrifuged at 10,000 rpm for 10 min at 4°C. The samples were concentrated in the dark via aeration of the solvent mixture using nitrogen gas, followed by the addition of 1.0 mL methanol and purification through a Sep-Pak^TM^ C_18_ reverse-phase extraction cartridge. The samples were dried completely, dissolved in 200 μL methanol, and filtered through a 0.22-mm PTFE filter. The IAA standards were prepared by dissolving IAA standards in methanol (Sigma, United States). Chromatographic and mass spectrometric conditions were as described previously (Niu et al., 2014). The experiment was performed in three biological and three technical replicates.

### Ultraperformance Liquid Chromatography-Tandem Mass Spectrometry (UPLC-MS/MS)

For the quantification of targeted hormones, flower samples were finely ground with liquid nitrogen following the protocol as described in Pan et al. (2010). Briefly, finely grounded flower samples and an adequate amount of internal standard (IS) were placed in 15 mL centrifuge tubes and 2-propanol/H_2_O/concentrated HCl (2:1:0.002, vol/vol/vol) were added to each tube followed by shaking at a speed of 5000 rpm for 10 min at 4°C. Thereafter, supernatants were transferred into a new tube and subjected under a gentle stream of highly purified nitrogen gas to a final volume of 3 mL and pH was adjusted to 8.0. Add twice the volume of petroleum ether to the solvent phase and shake at a speed of 5000 rpm for 10 min at 4°C and repeat this step. The sample solution was injected into the reverse-phase C_18_ Gemini HPLC column for UPLC-MS/MS analysis. The parameters of mass spectrometry for the measurement of hormones in the *H. coronarium* flowers are given in Supplementary Table 4. The experiment was performed in three biological and three technical replicates.

### GC-MS Analysis

To analyze volatile compounds, a flower was placed in a 250-mL glass bottle and covered with an aluminum sheet; ethyl caprate was used as an internal standard. After 30 min of incubation, a PDMS fiber was inserted into the bottle, incubated for 30 min to adsorb volatiles, and injected into a gas chromatography-mass spectrometry system (Agilent) for volatile analysis as described previously (Yue et al., 2015). The experiment was performed in five to seven biological replicates.

### Statistical Analysis

All data were analyzed using LSD with Origin software. *P*-values < 0.05 were considered to be significant.

## Data Availability Statement

The original contributions presented in the study are included in the article/Supplementary Material, further inquiries can be directed to the corresponding author/s.

## Author Contributions

YK, FA, and YF conceived and designed the experiment. YK, FA, and YZ contributed to the experiment and data analysis. YK and FA wrote the manuscript. FA, YF, and RY revised the manuscript. All authors contributed to the article and approved the submitted version.

## Conflict of Interest

The authors declare that the research was conducted in the absence of any commercial or financial relationships that could be construed as a potential conflict of interest.

## Publisher’s Note

All claims expressed in this article are solely those of the authors and do not necessarily represent those of their affiliated organizations, or those of the publisher, the editors and the reviewers. Any product that may be evaluated in this article, or claim that may be made by its manufacturer, is not guaranteed or endorsed by the publisher.

## Footnotes

1
https://wolfpsort.hgc.jp/

2
http://www.ncbi.nlm.nih.gov/

## Supplementary Material

Supplementary Figure 1 Phylogenetic analysis of six HcMYB proteins with *Arabidopsis* MYB protein family.

Supplementary Figure 2 Correlation between *HcMYB1* and *HcMYB2* expression and the emission of volatile compounds.

Supplementary Figure 3 A comparative analysis of transcript abundance of six *HcMYB* genes during flower development using RNA-seq data.

Supplementary Figure 4 The protein expression level of HcMYB1 and HcIAA4 in different tissues.

Supplementary Figure 5 The expression levels of key structural genes (*HcTPS5* and *HcBSMT2*) in different tissues.

Supplementary Figure 6 Heatmap showing expression profiles (log_2_TPM) of *HcBSMT* genes in different tissues.

Supplementary Figure 7 Heatmap showing expression profiles (log_2_TPM) of *HcTPS* genes in different tissues.

Supplementary Table 1 MYB-core binding motifs in the promoter regions of *HcBSMTs* and *HcTPSs*.

Supplementary Table 2 Primers used in this study.

Supplementary Table 3 Genes used for phylogenetic analysis and their accession numbers.

Supplementary Table 4 The parameters index of UPLC-MS/MS used for the measurement of hormones in the *H. coronarium* flowers.

## Abbreviations

AbA: aureobasidin A
ABA: abscisic acid
Aux/IAA: auxin/indole-3-acetic acid
BALD: benzaldehyde dehydrogenase
BiFC: bimolecular fluorescence complementation
BSMT: salicylic acid/benzoic acid methyltransferase
cDNA: complementary DNA
DMAPP: dimethylallyl pyrophosphate
FPP: farnesyl diphosphate
FPPS: FPP synthase
GC-MS: gas chromatography-mass spectrometer
GFP: green fluorescent protein
IAA: indole-3-acetic acid
NLS: nuclear localization signal
OD: optical density
PAL: phenylalanine ammonia lyase
SD: synthetically defined medium
TPS: terpene synthase
Y2H: yeast two-hybrid
Y1H: yeast one-hybrid.

## References

[B1] Abbas F. Ke Y. Yu R. Fan Y. (2019). Functional characterization and expression analysis of two terpene synthases involved in floral scent formation in *Lilium* ‘Siberia’. *Planta* 249 71–93. 10.1007/s00425-018-3006-7 30218384

[B2] Abbas F. Ke Y. Yu R. Yue Y. Amanullah S. Jahangir M. M. (2017). Volatile terpenoids: multiple functions, biosynthesis, modulation and manipulation by genetic engineering. *Planta* 246 803–816. 10.1007/s00425-017-2749-x 28803364

[B3] Abbas F. Ke Y. Zhou Y. Waseem M. Yu Y. Ashraf U. (2020). Cloning, functional characterization and expression analysis of *LoTPS5* from *Lilium* ‘Siberia’. *Gene* 756:144921. 10.1016/j.gene.2020.144921 32593719

[B4] Abbas F. Ke Y. Zhou Y. Yu Y. Waseem M. Ashraf U. (2021a). Genome-wide analysis of ARF transcription factors reveals HcARF5 expression profile associated with the biosynthesis of β-ocimene synthase in *Hedychium coronarium*. *Plant Cell Rep*. 40 1269–1284. 10.1007/s00299-021-02709-1 34052884

[B5] Abbas F. Ke Y. Zhou Y. Yu Y. Waseem M. Ashraf U. (2021b). Genome-wide analysis reveals the potential role of MYB transcription factors in floral scent formation in *Hedychium coronarium*. *Front*. *Plant Sci*. 12:623742. 10.3389/fpls.2021.623742 33719296 PMC7952619

[B6] Aharoni A. De Vos C. R. Wein M. Sun Z. Greco R. Kroon A. (2001). The strawberry *FaMYB1* transcription factor suppresses anthocyanin and flavonol accumulation in transgenic tobacco. *Plant J*. 28 319–332. 10.1046/j.1365-313x.2001.01154.x 11722774

[B7] An X. H. Tian Y. Chen K. Q. Liu X. J. Liu D. D. Xie X. B. (2014). MdMYB9 and MdMYB11 are Involved in the Regulation of the JA-Induced Biosynthesis of Anthocyanin and Proanthocyanidin in Apples. *Plant Cell Physiol.* 56 650–662. 10.1093/pcp/pcu205 25527830

[B8] Báez D. Pino J. A. Morales D. (2011). Floral scent composition in *Hedychium coronarium* J. Koenig analyzed by SPME. *J. Essent. Oil Res.* 23 64–67. 10.1080/10412905.2011.9700460

[B9] Bedon F. Bomal C. Caron S. Levasseur C. Boyle B. Mansfield S. D. (2010). Subgroup 4 R2R3-MYBs in conifer trees: gene family expansion and contribution to the isoprenoid-and flavonoid-oriented responses. *J*. *Exp*. *Bot*. 61 3847–3864. 10.1093/jxb/erq196 20732878 PMC2935864

[B10] Cao Y. Jia H. Xing M. Jin R. Grierson D. Gao Z. (2021). Genome-wide analysis of MYB gene family in Chinese bayberry (*Morella rubra*) and identification of members regulating flavonoid biosynthesis. *Front*. *Plant Sci*. 12:691384. 10.3389/fpls.2021.691384 34249063 PMC8264421

[B11] Chai Y. M. Jia H. F. Li C. L. Dong Q. H. Shen Y. Y. (2011). FaPYR1 is involved in strawberry fruit ripening. *J. Exp. Bot.* 62 5079–5089. 10.1093/jxb/err207 21778181

[B12] Colquhoun T. A. Schwieterman M. L. Wedde A. E. Schimmel B. C. Marciniak D. M. Verdonk J. C. (2011). *EOBII* controls flower opening by functioning as a general transcriptomic switch. *Plant Physiol*. 156 974–984. 10.1104/pp.111.176248 21464473 PMC3177291

[B13] Deluc L. Barrieu F. Marchive C. Lauvergeat V. Decendit A. Richard T. (2006). Characterization of a grapevine R2R3-MYB transcription factor that regulates the phenylpropanoid pathway. *Plant Physiol.* 140 499–511. 10.1104/pp.105.067231 16384897 PMC1361319

[B14] Deluc L. Bogs J. Walker A. R. Ferrier T. Decendit A. Merillon J. M. (2008). The transcription factor VvMYB5b contributes to the regulation of anthocyanin and proanthocyanidin biosynthesis in developing grape berries. *Plant Physiol*. 147 2041–2053. 10.1104/pp.108.118919 18539781 PMC2492604

[B15] Du H. Zhang L. Liu L. Tang X. F. Yang W. J. Wu Y. M. (2009). Biochemical and molecular characterization of plant MYB transcription factor family. *Biochemistry* 74 1–11. 10.1134/s0006297909010015 19232042

[B16] Dubos C. Le Gourrierec J. Baudry A. Huep G. Lanet E. Debeaujon I. (2008). MYBL2 is a new regulator of flavonoid biosynthesis in *Arabidopsis thaliana*. *Plant J.* 55 940–953. 10.1111/j.1365-313X.2008.03564.x 18532978

[B17] Dubos C. Stracke R. Grotewold E. Weisshaar B. Martin C. Lepiniec L. (2010). MYB transcription factors in *Arabidopsis*. *Trends Plant Sci.* 15 573–581. 10.1016/j.tplants.2010.06.005 20674465

[B18] Dudareva N. Klempien A. Muhlemann J. K. Kaplan I. (2013). Biosynthesis, function and metabolic engineering of plant volatile organic compounds. *New Phytol.* 198 16–32. 10.1111/nph.12145 23383981

[B19] Dudareva N. Negre F. Nagegowda D. A. Orlova I. (2006). Plant volatiles: recent advances and future perspectives. *Crit. Rev. Plant Sci.* 25 417–440. 10.1080/07352680600899973

[B20] Fan Y. P. Yu R. Huang Y. Chen Y. (2003). Studies on the essential constituent of *Hedychium flavum* and *H. coronarium*. *Acta Hortic. Sin.* 30:475. 10.16420/j.issn.0513-353x.2003.04.030

[B21] Fan Y.-P. Wang X.-R. Yu R.-C. Yang P. (2007). Analysis on the aroma components in several species of *Hedychium*. *Acta Hortic*. *Sin*. 34 231–234. 10.16420/j.issn.0513-353x.2007.01.049

[B22] Gershenzon J. Dudareva N. (2007). The function of terpene natural products in the natural world. *Nat. Chem. Biol.* 3 408–414. 10.1038/nchembio.2007.5 17576428

[B23] Hellens R. P. Allan A. C. Friel E. N. Bolitho K. Grafton K. Templeton M. D. (2005). Transient expression vectors for functional genomics, quantification of promoter activity and RNA silencing in plants. *Plant Methods* 1:13. 10.1186/1746-4811-1-13 16359558 PMC1334188

[B24] Hong G. J. Xue X. Y. Mao Y. B. Wang L. J. Chen X. Y. (2012). *Arabidopsis* MYC2 interacts with DELLA proteins in regulating sesquiterpene synthase gene expression. *Plant Cell* 24 2635–2648. 10.1105/tpc.112.098749 22669881 PMC3406894

[B25] Jaradat M. R. Feurtado J. A. Huang D. Lu Y. Cutler A. J. (2013). Multiple roles of the transcription factor AtMYBR1/AtMYB44 in ABA signaling, stress responses, and leaf senescence. *BMC Plant Biol.* 13:192. 10.1186/1471-2229-13-192 24286353 PMC4219380

[B26] Jia H. F. Chai Y. M. Li C. L. Lu D. Luo J. J. Qin L. (2011). Abscisic acid plays an important role in the regulation of strawberry fruit ripening. *Plant Physiol.* 157 188–199. 10.1104/pp.111.177311 21734113 PMC3165869

[B27] Jia H. Xie Z. Wang C. Shangguan L. Qian N. Cui M. (2017). Abscisic acid, sucrose, and auxin coordinately regulate berry ripening process of the Fujiminori grape. *Funct. Integr. Genomic* 17 441–457. 10.1007/s10142-017-0546-z 28224250

[B28] Jiang Y. Liang G. Yang S. Yu D. (2014). *Arabidopsis* WRKY57 functions as a node of convergence for jasmonic acid–and auxin-mediated signaling in jasmonic acid–induced leaf senescence. *Plant Cell* 26 230–245. 10.1105/tpc.113.117838 24424094 PMC3963572

[B29] Katiyar A. Smita S. Lenka S. K. Rajwanshi R. Chinnusamy V. Bansal K. C. (2012). Genome-wide classification and expression analysis of MYB transcription factor families in rice and *Arabidopsis*. *BMC Genomics* 13:544. 10.1186/1471-2164-13-544 23050870 PMC3542171

[B30] Ke M. Gao Z. Chen J. Qiu Y. Zhang L. Chen X. (2018). Auxin controls circadian flower opening and closure in the waterlily. *BMC Plant Biol.* 18:143. 10.1186/s12870-018-1357-7 29996787 PMC6042438

[B31] Ke Y. Abbas F. Zhou Y. Yu R. Yue Y. Li X. (2019). Genome-wide analysis and characterization of the Aux/IAA family genes related to floral scent formation in *Hedychium coronarium*. *Int. J. Mol. Sci.* 20:3235. 10.3390/ijms20133235 31266179 PMC6651449

[B32] Kim H. J. Park K. J. Lim J. H. (2011). Metabolomic analysis of phenolic compounds in buckwheat (*Fagopyrum esculentum* M.) sprouts treated with methyl jasmonate. *J. Agric. Food Chem.* 59 5707–5713. 10.1021/jf200396k 21417394

[B33] Kranz H. D. Denekamp M. Greco R. Jin H. Leyva A. Meissner R. C. (1998). Towards functional characterisation of the members of the R2R3−MYB gene family from *Arabidopsis thaliana*. *Plant J.* 16 263–276. 10.1046/j.1365-313x.1998.00278.x 9839469

[B34] Krizek B. A. (2011). Auxin regulation of *Arabidopsis* flower development involves members of the aintegumenta-like/plethora (AIL/PLT) family. *J. Exp. Bot.* 62 3311–3319. 10.1093/jxb/err127 21511900

[B35] Kumar S. Stecher G. Li M. Knyaz C. Tamura K. (2018). MEGA X: molecular evolutionary genetics analysis across computing platforms. *Mol. Biol. Evol.* 35 1547–1549. 10.1093/molbev/msy096 29722887 PMC5967553

[B36] Lai B. Du L. N. Liu R. Hu B. Su W. B. Qin Y. H. (2016). Two LcbHLH transcription factors interacting with LcMYB1 in regulating late structural genes of anthocyanin biosynthesis in *Nicotiana* and *Litchi chinensis* during anthocyanin accumulation. *Front*. *Plant Sci*. 7:166. 10.3389/fpls.2016.00166 26925082 PMC4757707

[B37] Lan J. B. Yu R. C. Yu Y. Y. Fan Y. P. (2013). Molecular cloning and expression of *Hedychium coronarium* farnesyl pyrophosphate synthase gene and its possible involvement in the biosynthesis of floral and wounding/herbivory induced leaf volatile sesquiterpenoids. *Gene* 518 360–367. 10.1016/j.gene.2013.01.007 23333605

[B38] Lavid N. Wang J. Shalit M. Guterman I. Bar E. Beuerle T. (2002). O-methyltransferases involved in the biosynthesis of volatile phenolic derivatives in rose petals. *Plant Physiol*. 129 1899–1907. 10.1104/pp.005330 12177504 PMC166779

[B39] Li J. Li X. Guo L. Lu F. Feng X. He K. (2006a). A subgroup of MYB transcription factor genes undergoes highly conserved alternative splicing in *Arabidopsis* and rice. *J. Exp. Bot.* 57 1263–1273. 10.1093/jxb/erj094 16531467

[B40] Li J. Yang X. Wang Y. Li X. Gao Z. Pei M. (2006b). Two groups of MYB transcription factors share a motif which enhances trans-activation activity. *Biochem. Biophys. Res. Commun.* 341 1155–1163. 10.1016/j.bbrc.2006.01.077 16460676

[B41] Li R. Fan Y. (2011). Molecular cloning and expression analysis of a terpene synthase gene, *HcTPS2*, in *Hedychium coronarium*. *Plant Mol. Biol. Rep.* 29 35–42. 10.1007/s11105-010-0205-1

[B42] Li Y. Shan X. Zhou L. Gao R. Yang S. Wang S. (2019). The R2R3-MYB factor FhMYB5 from *Freesia hybrida* contributes to the regulation of anthocyanin and proanthocyanidin biosynthesis. *Front. Plant Sci*. 9:1935. 10.3389/fpls.2018.01935 30666265 PMC6330306

[B43] Liao W. Yang Y. Li Y. Wang G. Peng M. (2016). Genome-wide identification of cassava R2R3 MYB family genes related to abscission zone separation after environmental-stress-induced abscission. *Sci. Rep.* 6:32006. 10.1038/srep32006 27573926 PMC5004182

[B44] Liu G. Ren G. Guirgis A. Thornburg R. W. (2009). The MYB305 transcription factor regulates expression of nectarin genes in the ornamental tobacco floral nectary. *Plant Cell* 21 2672–2687. 10.1105/tpc.108.060079 19783761 PMC2768911

[B45] Liu J. Osbourn A. Ma P. (2015). MYB transcription factors as regulators of phenylpropanoid metabolism in plants. *Mol*. *Plant* 8 689–708. 10.1016/j.molp.2015.03.012 25840349

[B46] Liu L. Ramsay T. Zinkgraf M. Sundell D. Street N. R. Filkov V. (2015). A resource for characterizing genome−wide binding and putative target genes of transcription factors expressed during secondary growth and wood formation in *Populus*. *Plant J.* 82 887–898. 10.1111/tpj.12850 25903933

[B47] Livak K. J. Schmittgen T. D. (2001). Analysis of relative gene expression data using real-time quantitative PCR and the 2(-Delta Delta C(T)) Method. *Methods* 25 402–408. 10.1006/meth.2001.1262 11846609

[B48] Luo J. Butelli E. Hill L. Parr A. Niggeweg R. Bailey P. (2008). AtMYB12 regulates caffeoyl quinic acid and flavonol synthesis in tomato: expression in fruit results in very high levels of both types of polyphenol. *Plant J.* 56 316–326. 10.1111/j.1365-313X.2008.03597.x 18643978

[B49] Medina-Puche L. Cumplido-Laso G. Amil-Ruiz F. Hoffmann T. Ring L. Rodríguez-Franco A. (2014). *MYB10* plays a major role in the regulation of flavonoid/phenylpropanoid metabolism during ripening of *Fragaria*× *ananassa* fruits. *J. Exp. Bot.* 65 401–417. 10.1093/jxb/ert377 24277278

[B50] Medina-Puche L. Molina-Hidalgo F. J. Boersma M. Schuurink R. C. López-Vidriero I. Solano R. (2015). An R2R3-MYB transcription factor regulates eugenol production in ripe strawberry fruit receptacles. *Plant Physiol.* 168 598–614. 10.1104/pp.114.252908 25931522 PMC4453772

[B51] Muhlemann J. K. Klempien A. Dudareva N. (2014). Floral volatiles: from biosynthesis to function. *Plant Cell Environ.* 37 1936–1949. 10.1111/pce.12314 24588567

[B52] Muhlemann J. K. Maeda H. Chang C. Y. San Miguel P. Baxter I. Cooper B. (2012). Developmental changes in the metabolic network of snapdragon flowers. *PLoS One* 7:e40381. 10.1371/journal.pone.0040381 22808147 PMC3394800

[B53] Niu Q. Zong Y. Qian M. Yang F. Teng Y. (2014). Simultaneous quantitative determination of major plant hormones in pear flowers and fruit by UPLC/ESI-MS/MS. *Anal. Methods* 6 1766–1773. 10.1039/c3ay41885E

[B54] Oono Y. Ooura C. Rahman A. Aspuria E. T. Hayashi K. I. Tanaka A. (2003). p-Chlorophenoxyisobutyric acid impairs auxin response in *Arabidopsis* root. *Plant Physiol*. 133 1135–1147. 10.1104/pp.103.027847 14526108 PMC281609

[B55] Pan X. Welti R. Wang X. (2010). Quantitative analysis of major plant hormones in crude plant extracts by high-performance liquid chromatography mass spectrometry. *Nat. Protoc.* 5 986–992. 10.1038/nprot.2010.37 20448544

[B56] Perkins-Veazie P. (1995). Growth and ripening of strawberry fruit. *Hortic. Rev.* 17 267–297. 10.1002/9780470650585.ch8

[B57] Pichersky E. Dudareva N. (2007). Scent engineering: toward the goal of controlling how flowers smell. *Trends Biotechnol*. 25 105–110. 10.1016/j.tibtech.2007.01.002 17234289

[B58] Qi T. Huang H. Wu D. Yan J. Qi Y. Song S. (2014). *Arabidopsis* DELLA and JAZ proteins bind the WD-repeat/bHLH/MYB complex to modulate gibberellin and jasmonate signaling synergy. *Plant Cell* 26 1118–1133. 10.1105/tpc.113.121731 24659329 PMC4001373

[B59] Raguso R. A. (2009). Floral scent in a whole−plant context: moving beyond pollinator attraction. *Funct*. *Ecol*. 23 837–840. 10.1111/j.1365-2435.2009.01643.x

[B60] Ramya M. Kwon O. K. An H. R. Park P. M. Baek Y. S. Park P. H. (2017). Floral scent: regulation and role of MYB transcription factors. *Phytochem. Lett.* 19 114–120. 10.1016/j.phytol.2016.12.015

[B61] Ramya M. Lee S. Y. An H. R. Park P. M. Kim N. S. Park P. H. (2019). MYB1 transcription factor regulation through floral scent in *Cymbidium* cultivar ‘Sael Bit’. *Phytochem. Lett.* 32 181–187. 10.1016/j.phytol.2019.06.007

[B62] Reddy V. A. Qian W. Dhar N. Kumar N. Venkatesh P. N. Rajan C. (2017). Spearmint R2R3−MYB transcription factor MsMYB negatively regulates monoterpene production and suppresses the expression of geranyl diphosphate synthase large subunit (*MsGPPS*. *LSU*). *Plant Biotechnol*. *J*. 15 1105–1119. 10.1111/pbi.12701 28160379 PMC5552485

[B63] Renner T. Bragg J. Driscoll H. E. Cho J. Jackson A. O. Specht C. D. (2009). Virus-induced gene silencing in the culinary ginger (*Zingiber officinale*): an effective mechanism for down-regulating gene expression in tropical monocots. *Mol*. *Plant* 2 1084–1094. 10.1093/mp/ssp033 19825682

[B64] Rommens C. M. Richael C. M. Yan H. Navarre D. A. Ye J. Krucker M. (2008). Engineered native pathways for high kaempferol and caffeoylquinate production in potato. *Plant Biotechnol. J.* 6 870–886. 10.1111/j.1467-7652.2008.00362.x 18662373

[B65] Rushton P. J. Somssich I. E. Ringler P. Shen Q. J. (2010). WRKY transcription factors. *Trends Plant Sci.* 15 247–258. 10.1016/j.tplants.2010.02.006 20304701

[B66] Shin B. Choi G. Yi H. Yang S. Cho I. Kim J. (2002). *AtMYB21*, a gene encoding a flower−specific transcription factor, is regulated by COP1. *Plant J.* 30 23–32. 10.1046/j.1365-313x.2002.01264.x 11967090

[B67] Shin R. Burch A. Y. Huppert K. A. Tiwari S. B. Murphy A. S. Guilfoyle T. J. (2007). The *Arabidopsis* transcription factor MYB77 modulates auxin signal transduction. *Plant Cell* 19 2440–2453. 10.1105/tpc.107.050963 17675404 PMC2002618

[B68] Sievers F. Wilm A. Dineen D. Gibson T. J. Karplus K. Li W. (2011). Fast, scalable generation of high−quality protein multiple sequence alignments using Clustal Omega. *Mol. Syst. Biol.* 7:539. 10.1038/msb.2011.75 21988835 PMC3261699

[B69] Spitzer-Rimon B. Farhi M. Albo B. Cna’ani A. Ben Zvi M. M. Masci T. (2012). The R2R3-MYB–like regulatory factor EOBI, acting downstream of EOBII, regulates scent production by activating *ODO1* and structural scent-related genes in petunia. *Plant Cell* 24 5089–5105. 10.1105/tpc.112.105247 23275577 PMC3556977

[B70] Spitzer-Rimon B. Marhevka E. Barkai O. Marton I. Edelbaum O. Masci T. (2010). *EOBII*, a gene encoding a flower-specific regulator of phenylpropanoid volatiles’ biosynthesis in petunia. *Plant Cell* 22 1961–1976. 10.1105/tpc.109.067280 20543029 PMC2910970

[B71] Tan H. Man C. Xie Y. Yan J. Chu J. Huang J. (2019). A crucial role of GA-regulated flavonol biosynthesis in root growth of *Araboidopsis*. *Mol*. *Plant* 12 521–537. 10.1016/j.molp.2018.12.021 30630075

[B72] Uimari A. Strommer J. (1997). Myb26: a MYB−like protein of pea flowers with affinity for promoters of phenylpropanoid genes. *Plant J.* 12 1273–1284. 10.1046/j.1365-313x.1997.12061273.x 9450341

[B73] Van Moerkercke A. Haring M. A. Schuurink R. C. (2011). The transcription factor emission of benzenoids II activates the MYB *ODORANT1* promoter at a MYB binding site specific for fragrant petunias. *Plant J.* 67 917–928. 10.1111/j.1365-313X.2011.04644.x 21585571

[B74] Verdonk J. C. Haring M. A. van Tunen A. J. Schuurink R. C. (2005). ODORANT1 regulates fragrance biosynthesis in petunia flowers. *Plant Cell* 17 1612–1624. 10.1105/tpc.104.028837 15805488 PMC1091778

[B75] Wu Q. Tao X. Ai X. Luo Z. Mao L. Ying T. (2018). Contribution of abscisic acid to aromatic volatiles in cherry tomato (*Solanum lycopersicum* L.) fruit during postharvest ripening. *Plant Physiol. Biochem.* 130 205–214. 10.1016/j.plaphy.2018.06.039 29990773

[B76] Yamaguchi N. Wu M. F. Winter C. M. Berns M. C. Nole-Wilson S. Yamaguchi A. (2013). A molecular framework for auxin-mediated initiation of flower primordia. *Dev. Cell* 24 271–282. 10.1016/j.devcel.2012.12.017 23375585

[B77] Yan H. Zhang H. Wang Q. Jian H. Qiu X. Wang J. (2011). Isolation and identification of a putative scent-related gene *RhMYB1* from rose. *Mol*. *Biol*. *Rep*. 38 4475–4482. 10.1007/s11033-010-0577-1 21132535

[B78] Yang Z. Li Y. Gao F. Jin W. Li S. Kimani S. (2020). MYB21 interacts with MYC2 to control the expression of terpene synthase genes in flowers of *Freesia hybrida* and *Arabidopsis thaliana*. *J*. *Exp*. *Bot*. 71 4140–4158. 10.1093/jxb/eraa184 32275056

[B79] Yoo S. D. Cho Y. H. Sheen J. (2007). *Arabidopsis* mesophyll protoplasts: a versatile cell system for transient gene expression analysis. *Nat. Protoc.* 2:1565. 10.1038/nprot.2007.199 17585298

[B80] Yoshida K. Oyama-Okubo N. Yamagishi M. (2018). An R2R3-MYB transcription factor ODORANT1 regulates fragrance biosynthesis in lilies (*Lilium* spp.). *Mol*. *Breed.* 38 1–14. 10.1007/s11032-018-0902-2

[B81] Yuan C. Li C. Yan L. Jackson A. O. Liu Z. Han C. (2011). A high throughput barley stripe mosaic virus vector for virus induced gene silencing in monocots and dicots. *PLoS One* 6:e26468. 10.1371/journal.pone.0026468 22031834 PMC3198768

[B82] Yue Y. Wang L. Yu R. Chen F. He J. Li X. (2021). Coordinated and high-level expression of biosynthetic pathway genes is responsible for the production of a major floral scent compound methyl benzoate in *Hedychium coronarium*. *Front. Plant Sci*. 12:650582. 10.3389/fpls.2021.650582 33897740 PMC8058416

[B83] Yue Y. Yu R. Fan Y. (2015). Transcriptome profiling provides new insights into the formation of floral scent in *Hedychium coronarium*. *BMC Genomics* 16:470. 10.1186/s12864-015-1653-7 26084652 PMC4472261

[B84] Zhang K. Logacheva M. D. Meng Y. Hu J. Wan D. Li L. (2018). Jasmonate-responsive MYB factors spatially repress rutin biosynthesis in *Fagopyrum tataricum*. *J. Exp. Bot.* 69 1955–1966. 10.1093/jxb/ery032 29394372 PMC6018783

[B85] Zhang X. He Y. Li L. Liu H. Hong G. (2021). Involvement of the R2R3-MYB transcription factors MYB21 and its homologs in regulating the stamen flavonols accumulation in *Arabidopsis*. *J*. *Exp*. *Bot*. 72 4319–4332. 10.1093/jxb/erab156 33831169 PMC8163065

[B86] Zhao Y. Xing L. Wang X. Hou Y. J. Gao J. Wang P. (2014). The ABA receptor PYL8 promotes lateral root growth by enhancing MYB77-dependent transcription of auxin-responsive genes. *Sci. Signal.* 7:ra53. 10.1126/scisignal.2005051 24894996 PMC4298826

[B87] Zhou M. Memelink J. (2016). Jasmonate-responsive transcription factors regulating plant secondary metabolism. *Biotechnol. Adv.* 34 441–449. 10.1016/j.biotechadv.2016.02.004 26876016

[B88] Zhou M. Zhang K. Sun Z. Yan M. Chen C. Zhang X. (2017). LNK1 and LNK2 corepressors interact with the MYB3 transcription factor in phenylpropanoid biosynthesis. *Plant Physiol.* 174 1348–1358. 10.1104/pp.17.00160 28483877 PMC5490896

[B89] Zhu N. Cheng S. Liu X. Du H. Dai M. Zhou D. X. (2015). The R2R3-type MYB gene *OsMYB91* has a function in coordinating plant growth and salt stress tolerance in rice. *Plant Sci.* 236 146–156. 10.1016/j.plantsci.2015.03.023 26025528

[B90] Zvi M. M. B. Shklarman E. Masci T. Kalev H. Debener T. Shafir S. (2012). PAP1 transcription factor enhances production of phenylpropanoid and terpenoid scent compounds in rose flowers. *New Phytol.* 195 335–345. 10.1111/j.1469-8137.2012.04161.x 22548501

[B91] Zou X. Neuman D. Shen Q. J. (2008). Interactions of two transcriptional repressors and two transcriptional activators in modulating gibberellin signaling in aleurone cells. *Plant Physiol.* 148 176–186. 10.1104/pp.108.123653 18621977 PMC2528090

