# Supplementary material for: Auxin-Responsive R2R3-MYB Transcription Factors HcMYB1 and HcMYB2 Activate Volatile Biosynthesis in Hedychium coronarium Flowers
Source: Front Plant Sci. 2021 Aug 3;12:710826. doi: 10.3389/fpls.2021.710826 (PMC8369990; doi:10.3389/fpls.2021.710826)
Supplement: Supplementary Figure 1 — Phylogenetic analysis of six HcMYB proteins with Arabidopsis MYB protein family. [file Data_Sheet_1.PDF]

Table S1: MYB-core binding motifs present in the promoter regions of *HcBSMTs* and *HcTPSs*.

**Promoter of BSMTs**	**MYB-Core motif (C/T)NGTT(A/G) Start position & Strand**
Pro-HcBSMT2 (1131 bp)	-191(+), -230(-), -294(-), -300(+),-326(+), -557(+), -622(-),-809(+), -879(-), -878(+), -906(+), -968(-),-1028(+)
Pro-evm.model.scaf_102.154	-8(+), -451(+), -810(+), -897(-), -1828(-)
Pro-evm.model.scaf_368.25	-488(+)
Pro-evm.model.scaf_368.54	-50(-), -601(-), -1263(-), -1430(+)
Pro-evm.model.scaf_397.41	-807(+), -900(-), -1474(+)
Pro-evm.model.scaf_397.42	-314(-), -489(-), -545(-), -573(-), -1742(-), -1835(-)
Pro-evm.model.scaf_462.93	-400(-), -424(+), -1089(+), -1457(+), -1577(-), -1745(+)
Pro-evm.model.scaf_68.67	-1643(+), -1875(+), -1995(-)
Pro-evm.model.scaf_68.69	-861(-)
Pro-evm.model.scaf_72.107	-96(-), -312(+), -1965(-)
**Promoter of TPSs**	
Pro-HcTPS5 (1555 bp)	-366(+), -1178(+), -1169(+),-1208(+), -1310(+)
Pro-evm.model.scaf_1065.6	-756(+), -832(+), -1440(+)
Pro-evm.model.scaf_116.98	-1423(-), -1520(+), -1571(-), -1800(+)
Pro-evm.model.scaf_1294.1	-1190(-), -1246(+), -1335(-)
Pro-evm.model.scaf_158.171	-63(+), -309(+), -562(+), -602(-)
Pro-evm.model.scaf_162.50	-10(-), -144(-), -223(-), -489(-)
Pro-evm.model.scaf_174.2	-139(-), -461(+), -1052(+), -1061(+), -1091(+), -1193(+)
Pro-evm.model.scaf_182.35	-950(+), -1010(-), -1423(-), -1767(+)
Pro-evm.model.scaf_192.73	-474(+), -1286(+), -1495(-), -1911(+)
Pro-evm.model.scaf_192.74	-58(-), -949(-), -1487(+)
Pro-evm.model.scaf_206.89	-290(-), -508(+), -1048(+), -1160(+), -1245(+), -1780(+)

Pro-evm.model.scaf_206.91	-2(-), -1927(+)
Pro-evm.model.scaf_206.94	-404(+), -609(+), -835(+), -218(+), -1027(-), -1211(-), -1559(+), -
Pro-evm.model.scaf_206.95	-349(-), -106(+), -1989(-)
Pro-evm.model.scaf_209.2	-678(-), -1341(-), -1802(+)
Pro-evm.model.scaf_257.59	-983(-), -1598(-)
Pro-evm.model.scaf_257.61	-560(+), -915(+), -1452(-),
Pro-evm.model.scaf_285.47	-1476(-), -1764(+)
Pro-evm.model.scaf_291.36	-68(-), -72(-), -149(-), -661(+), -1318(+), -1837(-)
Pro-evm.model.scaf_291.37	-812(+), -716(+), -1771(+)
Pro-evm.model.scaf_291.39.1	-368(+), -1171(+), -1180(+), -1210(+), -1312(+)
Pro-evm.model.scaf_291.43	-158(+), -190(+), -289(+), -622(+), -1216(+), -1863(+)
Pro-evm.model.scaf_324.6	-43(-), -296(+), -326(+), -485(+), -518(+), -822(+), -847(-)
Pro-evm.model.scaf_345.47	-59(+), -444(-), -1044(-), -1890(+)
Pro-evm.model.scaf_430.18	-324(-), -375(-), -669(+), -1426(+), -1493(-), -1636(-), -1788(-)
Pro-evm.model.scaf_430.22	-1106(+), -1773(+)
Pro-evm.model.scaf_457.3	-29(-), -235(-), -449(-), -535(+), -844(+), -1154(+), -1500(-), -
Pro-evm.model.scaf_48.3	-841(+), -1164(-)
Pro-evm.model.scaf_509.28	-548(+), -756(-), -1810(-)
Pro-evm.model.scaf_544.1	-301(-), -809(-), -1239(-), -1569(+)
Pro-evm.model.scaf_544.13	-445(+), -1570(-), -1653(+), -1689(-), -1718(-), -1793(-)
Pro-evm.model.scaf_544.15	-225(-), -423(-), -536(+), -1137(+), -1485(+), -1515(+), -1522(+), -
Pro-evm.model.scaf_544.17	-239(+), -976(-), -1615(+), -1646(-)
Pro-evm.model.scaf_544.4	-366(+),
Pro-evm.model.scaf_568.16	-99(+), -709(+)
Pro-evm.model.scaf_568.17	-273(-), -658(-), -754(+), -956(+), -977(+)
Pro-evm.model.scaf_568.19	-45(-), -365(-), -437(-), -450(+), -644(-), -692(-), -963(+)
Pro-evm.model.scaf_568.23	-277(-), -820(+), -1958(+),
Pro-evm.model.scaf_625.34	-66(-), -225(+), -496(+), -819(-), -1128(+), -1321(+), -1368(-), -
Pro-evm.model.scaf_625.36	-13(-), -707(+), -1823(-)
Pro-evm.model.scaf_625.40	-949(-), -1027(-), -1277(+), -1372(-), -1469(-), -1473(-)
Pro-evm.model.scaf_625.43	-68(+), -644(-), -1648(-), -1820(-)

Pro-evm.model.scaf_625.47	-187(-), -222(-), -392(-), -783(+), -845(-), -896(+), -1779(+)
Pro-evm.model.scaf_636.15	-578(+), -762(-), -778(+), -782(+), -1060(-), -1540(+)
Pro-evm.model.scaf_64.7	-119(+), -212(-), -243(+), -1492(+), -1515(+)
Pro-evm.model.scaf_64.8	-64(-), -104(-), -295(+), -804(+), -1545(+), -1900(+)
Pro-evm.model.scaf_666.24	-3(+), -177(-), -295(-), -429(-), -624(-), -895(-), -1536(+)
Pro-evm.model.scaf_71.94_evm.model.scaf_71.95	-771(+), -938(+), -1456(-), -1788(+), -1794(+)
Pro-evm.model.scaf_74.108	-270(+), -340(-), -780(-), -1284(-)
Pro-evm.model.scaf_79.34.5	-852(-), -886(-), -1091(-), -1362(+)
Pro-evm.model.scaf_79.35	-214(-), -1383(-), --1581(+), 1880(-)
Pro-evm.model.scaf_802.2	-51(+), -1952(+)
Pro-evm.model.scaf_893.1	-161(+), -209(+), -893(+), -1157(+), -1449(+), -1687(-), -1638(+)
Pro-evm.model.scaf_893.20	-814(+), -181(-), -1019(-), -1948(+),
Pro-evm.model.scaf_893.21	-346(+), -689(-)
Pro-evm.model.scaf_9.60	-192(+), -614(-), -806(+), -991(-), -1028(-), -1478(+), -1525(-), -
Pro-evm.model.scaf_9.62	-1184(-), -1379(-), -1935(+), -1945(+)
Pro-evm.model.scaf_923.3	-277(-), -311(-), -524(-), -632(+), -805(-), -840(-), -889(+), -1810(-)
Pro-evm.model.scaf_96.105	-51(+), -668(+), -767(+), -1965(+)
Pro-evm.model.scaf_96.107	-12(+), -264(-), -270(+)

Table S2: Primers used in the experiments

**Cloning primers**		Upstream / downstream primers （5′-3′）
HcMYB1	F	TGTCTCCTTGCTTGCGGCAAC
	R	CAAGCCCATGCAATGGCAAAATCG
HcMYB2	F	CTGTCTCTCACAATCCCTCTAT
	R	ATTCTCCATCTTAGTCACTTGC
HcBSMT2	F	GCCTAGGATGCTCATTGAGTGAC
	R	CTTGCTCCACCTTCAAACCCAT
**qRT-PCR primers**		
HcMYB1	F	GTCAGTAGCAGCACCAGC
	R	GCAGAGACGAAGCCCAAGAT
HcMYB2	F	GAGGCAGATGCGGTGTGAC

	R	CTCATCCATCCAGCCGCAA
HcBSMT2	F	AGGAGAAAGCCAATCACACC
	R	GGCATGATTCAGTTTGACAAG
HcTPS5	F	ATTACTTGCGGTGTTCCTGCTG
	R	AACGAGCCATCCCTCCCATT
**BSMV**		
HcMYB1	F	AAGGAAGTTTAAACCGACAACGACATCAAGA
	R	AACCACCACCACCGTCAGTCCCATCCAACCCAT
HcMYB2	F	AAGGAAGTTTAACCCGAGTGCA GAAGCACGC
	R	AACCACCACCACCGTCTCATCCAT CCAGCCGCAA
**GAPDH**		
	F	GGTATTGTCGAGGGTTTGATG
	R	GCTGTTGGCAAAGTTCTCCCT
**GFP primers**		
HcMYB1	F	CAAATTCGCGACCGGT ATGGGGAGGGCTCCTTG
	R	TGCTAGTCATACCGGT GAGCTGCTGTTGTAACC
HcMYB2	F	CAAATTCGCGACCGGT ATGGAGTTCCACGGGAGG
	R	TGCTAGTCATACCGGT GTTCAAAAGATTCATCG
**Protein-GST**		
HcMYB1	F	GAATTCCCGGGTCGAC ATGGGGAGGGCTCCTTG
	R	GGCCGCTCGAGTCGAC GAGCTGCTGTTGTAACC
HcMYB2	F	GAATTCCCGGGTCGAC ATGGAGTTCCACGGGAGG
	R	GGCCGCTCGAGTCGAC GTTCAAAAGATTCATCG
**pBD**		
HcMYB1	F	TCGCCGACCGGTAGGCCT ATGGGGAGGGCTCCTTG
	R	AACCAGAGTTAAAGGCCT CAGTCGGCAATGGTACA
HcMYB2	F	TCGCCGACCGGTAGGCCT ATGGAGTTCCACGGGAGG
	R	AACCAGAGTTAAAGGCCT TTAGAGCTGCTGTTGTAACC
**pGADT7 （AD）**		
HcMYB1	F	TCGCCGACCGGTAGGCCT ATGGGGAGGGCTCCTTG
	R	AACCAGAGTTAAAGGCCT CAGTCGGCAATGGTACA
HcMYB2	F	TCGCCGACCGGTAGGCCT ATGGAGTTCCACGGGAGG
	R	AACCAGAGTTAAAGGCCT TTAGAGCTGCTGTTGTAACC
**62SK for dual-Luciferase**		
HcMYB1	F	TGCAGGAATT CGATATC AAGCTT ATGGGGAGGGCTCCTTG
	R	ATTTCAGCGTACCGAATT GGTACC CTACAGTCGGCAATGGTACA
HcMYB2	F	TGCAGGAATT CGATATC AAGCTT

		ATGGAGTTCCACGGGAGG
	R	ATTTCAGCGTACCGAATT GGTACC TTAGAGCTGCTGTTGTAACC
**0800 for dual-Luciferase**		
HcBSMT2	F	GGTCGAC GGTATCGAT AAGCTT GATGCTCATTGAGTGACAAAT
	R	CCGCTCTAGAACTAGT GGATCC CTTTCTCCCTCTCTCTCA
**Primers for pAbAi**		
HcBSMT2	F	CCAAGCTT TCAAAGGTTTCACACACAAATC
	R	GGGGTACC CCGGCAAAAAAAAAAGTTTACAC
**Primers for probe**		
proHcBSMT2	F	CAAGCGACCACCAACGGTTGAGATCAATTTTTTCTTGATCTAACGGTTC
	R	GAACCGTTAGATCAAGAAAAAATTGATCTCAACCGTTGGTGGTCGCTTG
ProHcTPS5	F	TTCAACCAGATCAACCAATATATAACTCCTTAGCCCCTACCTAACCAGAC
	R	GTCTGGTTAGGTAGGGGCTAAGGAGTTATATATTGGTTGATCTGGTTGAA

Table S3: Genes used in phylogenetic tree and their accession numbers.

No.	Gene name	Plant Name	Accession number
1	FaEOBII	*Fragaria x ananassa*	KM099230
2	AmMYB305	*Antirrhinum majus*	P81391
3	AmMYB340	*Antirrhinum majus*	P81396
4	PsMYB26	*Pisum sativum*	Y11105
5	PhEOBII	*Petunia hybrida*	EU360893
6	NlMYB305	*Nicotiana langsdorffii*	EU111679
7	FaMYB1	*Fragaria x ananassa*	AF401220
8	FaMYB10	*Fragaria x ananassa*	EU155162
9	AtMYB11	*Arabidopsis thaliana*	AT3G62610
10	AtMYB12	*Arabidopsis thaliana*	AT2G47460
11	AtMYB123	*Arabidopsis thaliana*	AT5G35550
12	AtMYB111	*Arabidopsis thaliana*	AT5G49330

13	AtMYB113	*Arabidopsis thaliana*	AT1G66370
14	AtMYB114	*Arabidopsis thaliana*	AT1G66380
15	AtMYB21	*Arabidopsis thaliana*	AT3G27810
16	AtMYB24	*Arabidopsis thaliana*	AT5G40350
17	AtMYB2	*Arabidopsis thaliana*	AT2G47190
18	AtMYB108	*Arabidopsis thaliana*	AT3G06490
19	AtMYB78	*Arabidopsis thaliana*	AT5G49620
20	AtMYB122	*Arabidopsis thaliana*	AT1G74080
21	AtMYB51	*Arabidopsis thaliana*	AT1G18570
22	AtMYB34	*Arabidopsis thaliana*	AT5G60890
23	AtMYB29	*Arabidopsis thaliana*	AT5G07690
24	AtMYB76	*Arabidopsis thaliana*	AT5G07700
25	AtMYB28	*Arabidopsis thaliana*	AT5G61420
26	AtMYB38	*Arabidopsis thaliana*	AT2G36890
27	AtMYB37	*Arabidopsis thaliana*	AT5G23000
28	AtMYB77	*Arabidopsis thaliana*	AT3G50060
29	AtMYB44	*Arabidopsis thaliana*	AT5G67300

Table S4: The determination of mass spectrometry for measure endogenous hormones in the petals of *H. coronarium*

Compound name	ESI mode	Parent(m/z)	Daughter	Dwell(s)	Cone(V)	Collision
**IAA**	ESI+	175.900	102.967	0.029	24	28
**JA**	ESI-	209.000	59.000	0.082	26	12
**ABA**	ESI-	263.000	153.000	0.029	25	9

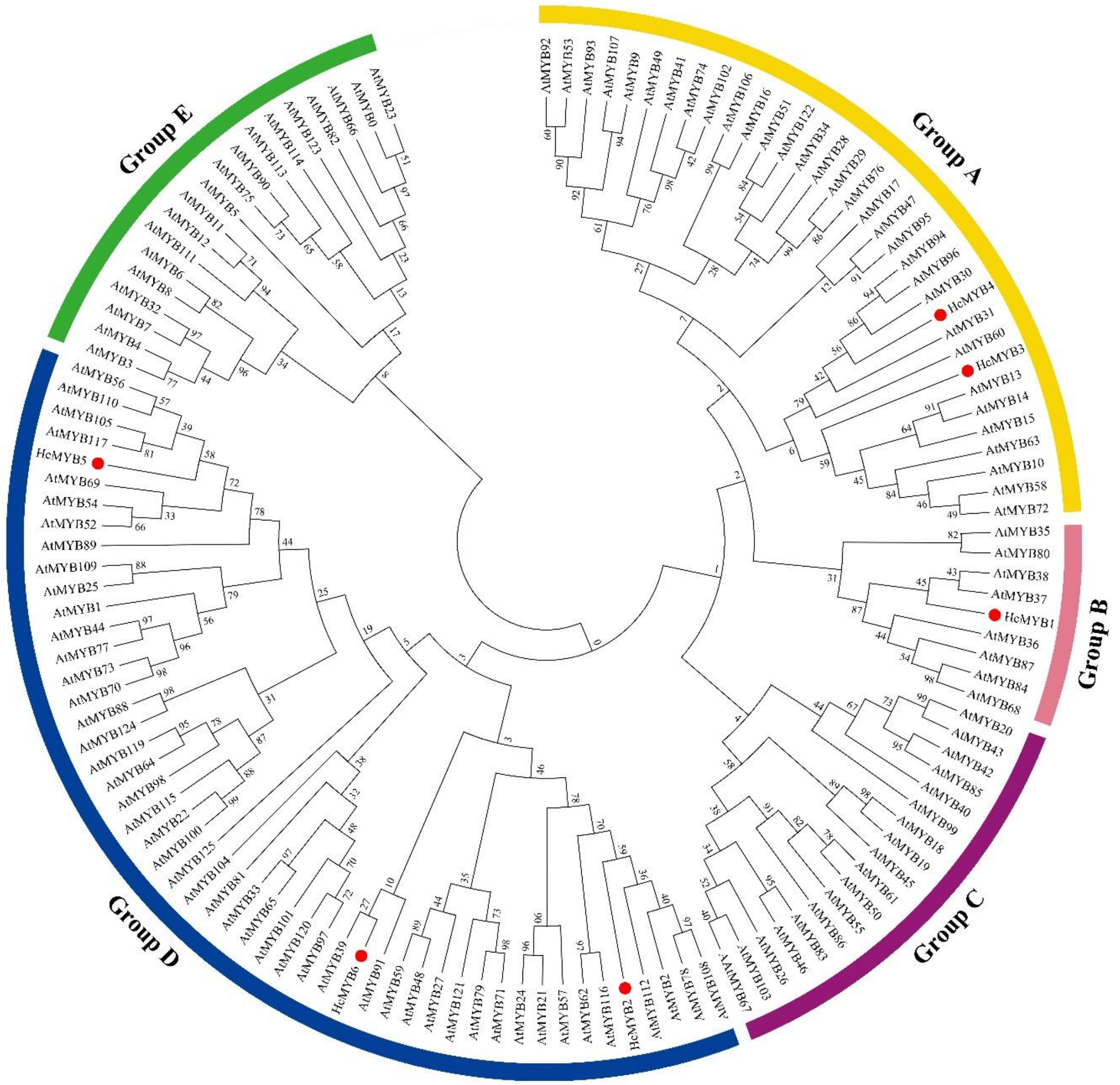

Figure S1: Phylogenetic analysis of six HcMYB proteins with *Arabidopsis* MYB protein family.

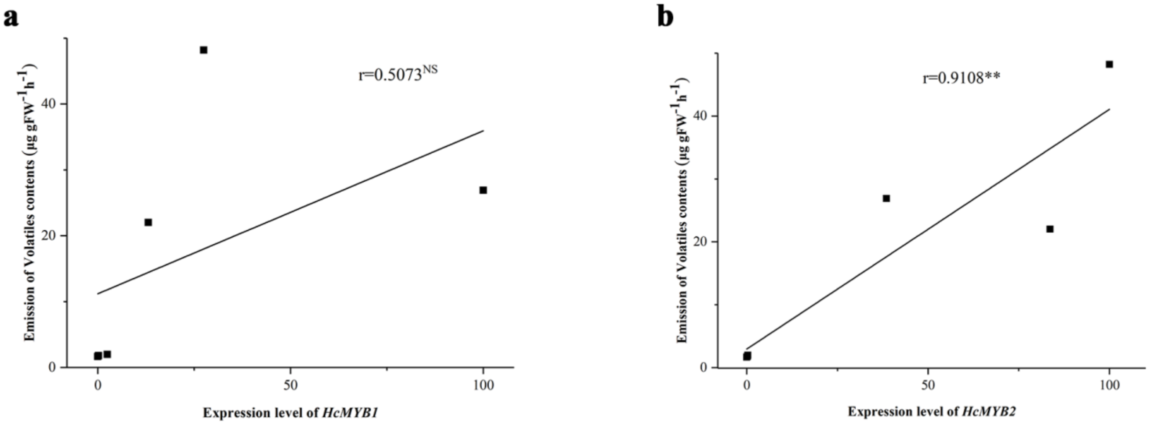

Figure S2: Correlation between the expression of HcMYB1 and HcMYB2 with the emission of volatile contents.

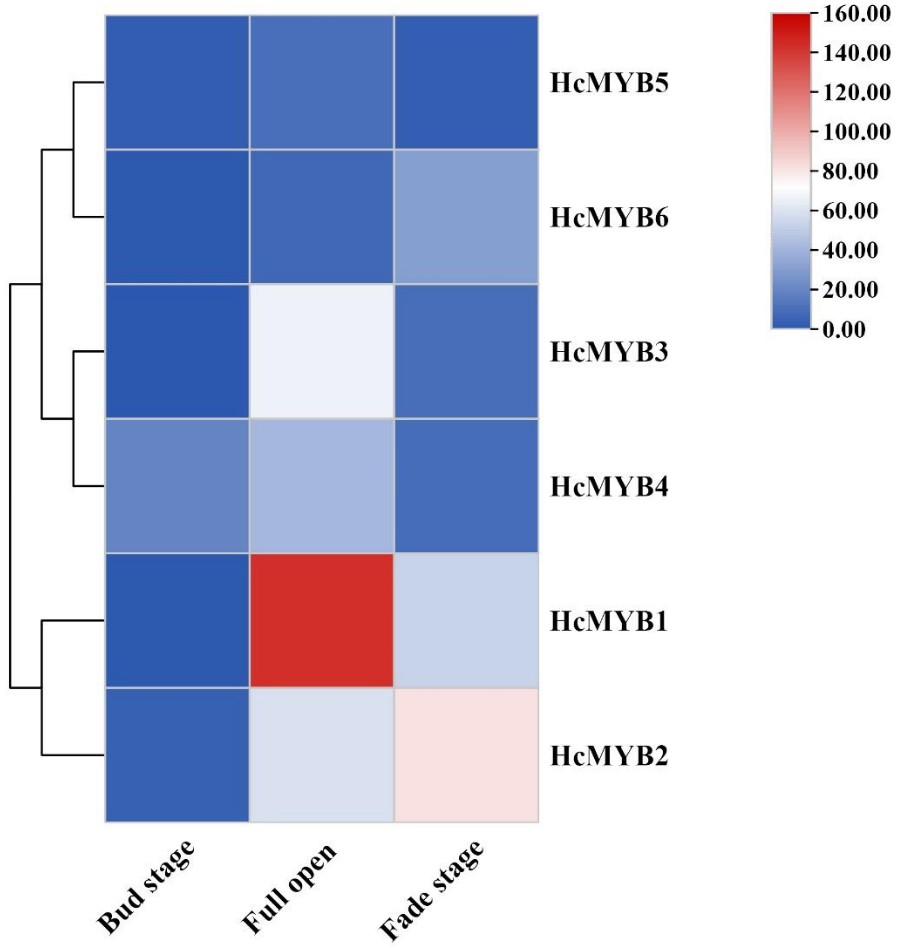

Figure S3: A comparative analysis of transcript abundance of six *HcMYB* genes during flower development using RNA-seq data.

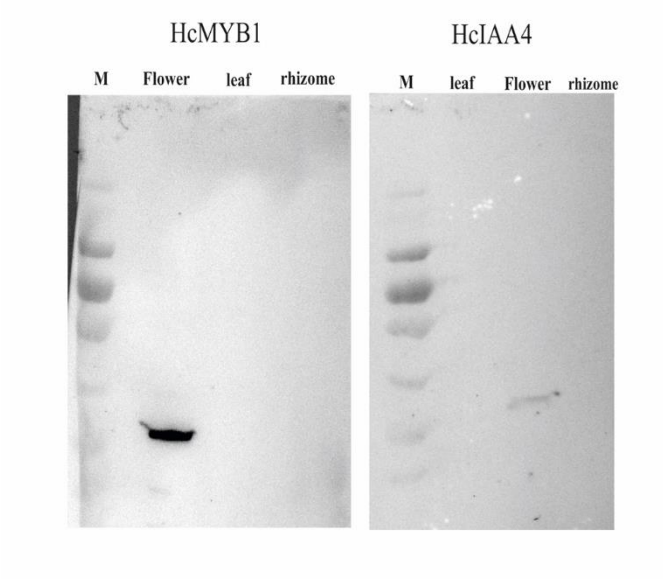

Figure S4: The protein expression level of HcMYB1 and HcIAA4 in different tissues.

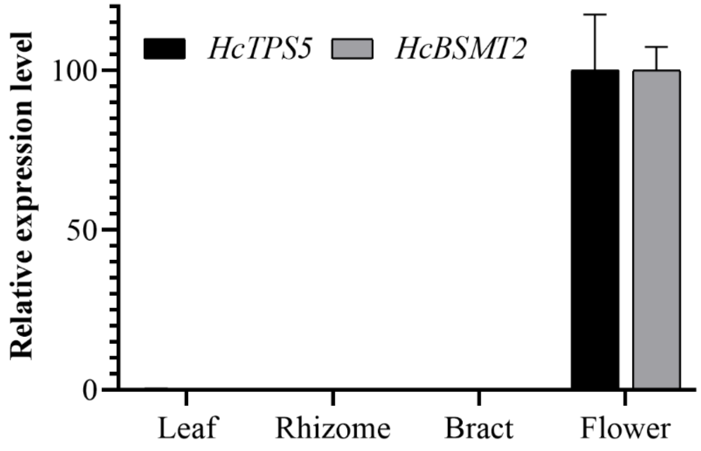

Figure S5: The expression levels of key structural genes (*HcTPS5* and *HcBSMT2*) in different tissues.

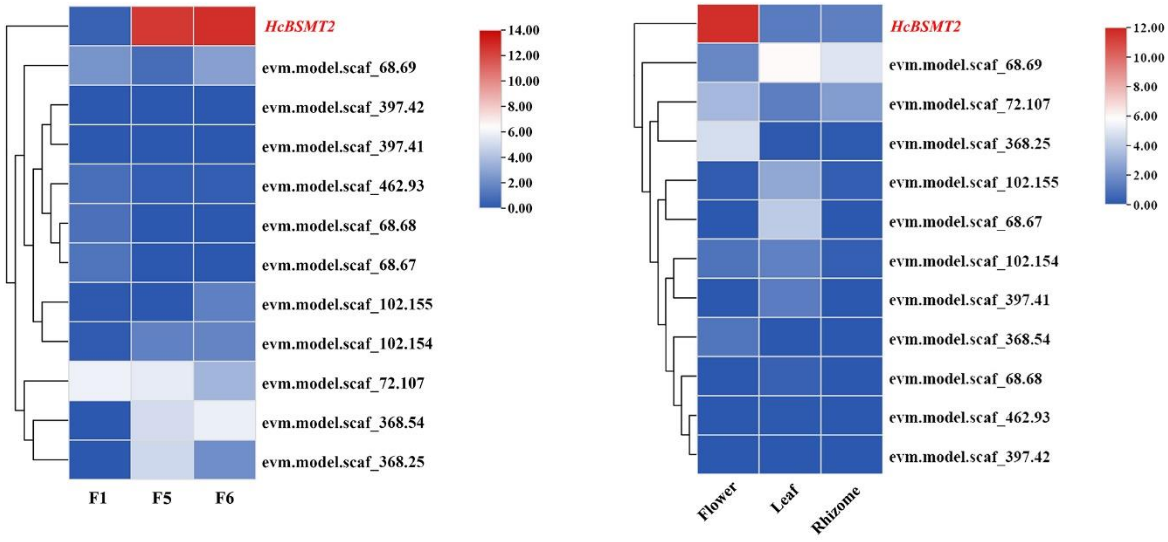

Figure S6: Heatmap showing expression profiles ($\log_2$TPM) of *HcBSMT* genes in different tissues.

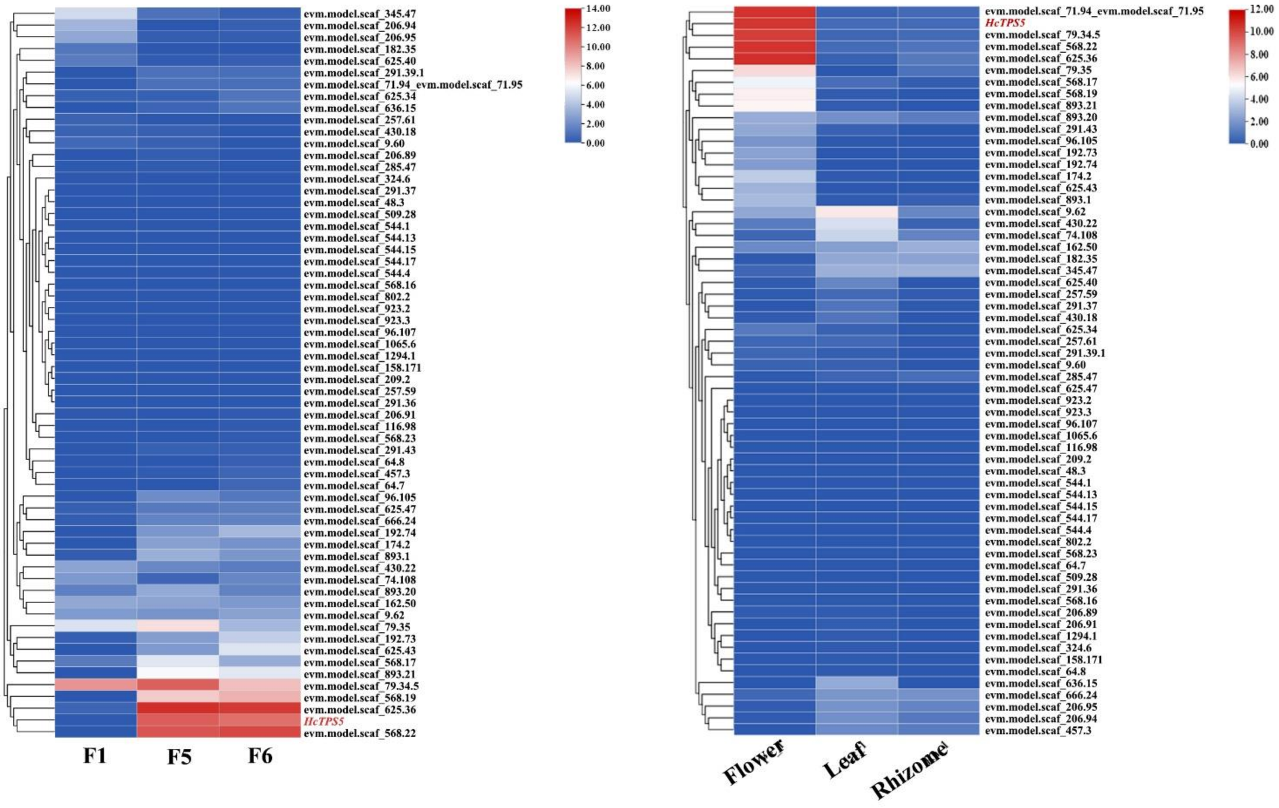

Figure S7: Heatmap showing expression profiles ($\log_2$TPM) of *HcTPS* genes in different tissues.
